# Androgen Receptor-Target Genes in African American Prostate Cancer Disparities

**DOI:** 10.1155/2013/763569

**Published:** 2013-01-10

**Authors:** Bi-Dar Wang, Qi Yang, Kristin Ceniccola, Fernando Bianco, Ramez Andrawis, Thomas Jarrett, Harold Frazier, Steven R. Patierno, Norman H. Lee

**Affiliations:** ^1^Department of Pharmacology and Physiology, The George Washington University Medical Center, Washington, DC 20037, USA; ^2^Medical Faculty Associates, The George Washington University Medical Center, Washington, DC 20037, USA; ^3^Division of Urology, Mount Sinai Medical Center, Columbia University, Miami Beach, FL 33140, USA; ^4^The GW Cancer Institute, The George Washington University Medical Center, Washington, DC 20037, USA; ^5^Duke Cancer Institute, Duke University Medical Center, Durham, NC 27710, USA

## Abstract

The incidence and mortality rates of prostate cancer (PCa) are higher in African American (AA) compared to Caucasian American (CA) men. To elucidate the molecular mechanisms underlying PCa disparities, we employed an integrative approach combining gene expression profiling and pathway and promoter analyses to investigate differential transcriptomes and deregulated signaling pathways in AA versus CA cancers. A comparison of AA and CA PCa specimens identified 1,188 differentially expressed genes. Interestingly, these transcriptional differences were overrepresented in signaling pathways that converged on the androgen receptor (AR), suggesting that the AR may be a unifying oncogenic theme in AA PCa. Gene promoter analysis revealed that 382 out of 1,188 genes contained *cis*-acting AR-binding sequences. Chromatin immunoprecipitation confirmed *STAT1, RHOA, ITGB5, MAPKAPK2, CSNK2A,1* and *PIK3CB* genes as novel AR targets in PCa disparities. Moreover, functional screens revealed that androgen-stimulated AR binding and upregulation of *RHOA, ITGB5,* and *PIK3CB* genes were associated with increased invasive activity of AA PCa cells, as siRNA-mediated knockdown of each gene caused a loss of androgen-stimulated invasion. In summation, our findings demonstrate that transcriptional changes have preferentially occurred in multiple signaling pathways converging (“transcriptional convergence”) on AR signaling, thereby contributing to AR-target gene activation and PCa aggressiveness in AAs.

## 1. Introduction

 Prostate cancer (PCa) is the most commonly diagnosed noncutaneous cancer and, after lung and bronchus cancers, the second leading cause of cancer deaths among American men [1, 2]. In the United States, it is estimated that 241,740 men will be newly diagnosed with prostate cancer, and 28,170 will succumb to this disease in 2012 (http://www.cancer.gov/cancertopics/types/prostate).

The human androgen receptor (AR) plays a critical role in the growth and differentiation of the normal prostate gland as well as in the development of PCa [3, 4]. AR expression has been observed in nearly all primary PCa cases [5–7]. Previous studies have also shown that the cellular AR levels are correlated to primary and metastatic lesions and associated with disease progression to castration-resistant PCa (CRPCa) [8–10]. 

 In the US, the African American (AA) population exhibits higher incidence and mortality rates of PCa compared to the Caucasian American (CA) population [11]. Accumulating evidence has suggested that biological factors may in part play a critical role in PCa health disparities that is observed among racial groups. The AR signaling pathway has been implicated as one of critical biological mechanisms associated with PCa disparities. For instance, it has been reported that AA men have higher mean serum testosterone levels compared to CA men [12, 13]. Furthermore, the expression of AR protein is 22% higher in benign and 81% higher in malignant prostate tissues of AA patients compared to their CA counterparts undergoing radical prostatectomy [14]. Genetic mutations contributing to higher serum dihydrotestosterone to testosterone ratios have been forwarded as another mechanism underlying PCa disparities. Thymine-Adenine (TA) dinucleotide repeat and A49T variants of the SRD5A2 gene, which encodes a type II 5-*α*-reductase, are prevalent in AA men. In addition, these two polymorphisms were shown to correlate with the elevated 5-*α*-reductase activity [15] and higher conversion rate of testosterone to dihydrotestosterone (DHT) [16], respectively, in AA patients. The architecture of the AR gene has also provided hints into the mechanism of the PCa health disparities. Exon 1 of the AR gene, encoding the N-terminal transactivation domain, was found to have two polymorphic trinucleotide repeats (CAG and GGC, the codons for glutamine and glycine, resp.) [17]. The CAG repeat length is inversely correlated with AR transcriptional activity [18], and previous reports have revealed that AA men tend to have significantly shorter CAG repeats than CA men [17, 19, 20]. The shorter CAG and GGC repeats have been associated with a higher risk for developing PCa [21–23]. Taken together, these findings suggest that differences in androgenic activities between AA and CA populations may play an important role in PCa health disparities.

Gene expression analysis by DNA microarrays, and more recently by next generation sequencing, has proven to be a useful tool in assigning genomic signatures to different subtypes and stages of cancers. The goal of these analyses has been to provide more precise diagnoses and improved predictions of clinical outcomes. To date, the majority of PCa microarray studies have been employed in the comparison of gene expression profiles in normal prostate and different stages of PCa [24–31], without consideration of patient racial background. Only a handful of microarray studies have been initiated to address the molecular underpinnings of PCa disparities [32, 33]. Despite the recognized role of AR in PCa disparities, no microarray studies have been conducted to specifically address the genomics of AR signaling in AA PCa, albeit an earlier study used a microarray approach to identify androgen-regulated genes as a means for biomarker discovery in PCa in the general population [34].

In this study, we have employed DNA microarrays and bioinformatics to compare the gene expression profiles between AA and CA cancers, and between PCa and patient-matched normal prostate from AAs and CAs. By integrating gene expression profiling and pathway analyses, multiple components within the AR signaling pathway were revealed to be upregulated in AA PCa specimens (along with the up-regulation of genes within other signaling pathways that converge on AR signaling), portending that AR pathway activation is a key component of PCa health disparities. The upregulated AR signaling pathway was associated with 382 AR-responsive genes based on microarray and promoter analyses; selected AR-responsive genes were confirmed by chromatin immunoprecipitation and functionally validated to participate in AR-mediated invasive activity in AA PCa cell lines. These results provide a first glimpse of AR-responsive genes likely contributing to the more aggressive PCa phenotype in AA men.

## 2. Materials and Methods

### 2.1. Acquisition of Patient Tissue Specimens

Prostate tissue procurement procedures implemented at The George Washington University Medical Faculty Associates (GWU-MFA) were in accordance with IRB approved protocols. A transrectal ultrasound biopsy of the prostate was performed in patients with an elevated serum PSA level of >7 ng/mL, or PSA level of >4 ng/mL in the presence of an abnormal digital rectal exam. Prostate needle biopsy cores were collected and immediately examined by a board certified pathologist at the GWU-MFA. PCa cores eligible for genomic analysis were determined to have a pathologic tumor stage of 2 and Gleason score of 6-7 (17 AA and 13 CA patients) or 8-9 (3 AA and 2 CA patients). There was no significant difference between the two racial groups with respect to age (average age for AAs and CAs was 62.3 ± 8.2 and 63.3 ± 9.2, resp.). In addition, there was no significant difference between the two groups with respect to tumor content in the biopsies (average percentages of tumor for AA and CA biopsies were 52.25 ± 9.38% and 47.44 ± 4.20%, resp.). Paired normal biopsy cores were also collected from the same patients. Altogether in this study, each of the 20 AA and 15 CA patients contributed a PCa biopsy core along with a patient-matched normal core for DNA microarray analysis and qRT-PCR validation.

### 2.2. Microarray Analysis and Bioinformatics

Total RNA was isolated from PCa and patient-matched normal prostate biopsy cores. Total RNA (~1 *μ*g) from each core was purified using the RNeasy micro kit (Qiagen, Valencia, CA, USA) and interrogated for mRNA expression patterns with the Affymetrix Human Exon 1.0 ST GeneChip. High-quality, non-degraded total RNA was confirmed on the Agilent 2100 Bioanalyzer prior to performing expression profiling experiments. Affymetrix microarray data were normalized by quantile normalization with GC-RMA background correction, and data visualization and statistical analysis were performed by Partek Genomics Suite 6.5 software (Partek, St. Louis, MO, USA) as described previously [35]. Statistical analysis of microarray data comparing AA cancer versus CA cancer, CA cancer versus patient-matched normal tissue, and AA cancer versus patient-matched normal tissue was performed based on ANOVA with a 10% False Discovery Rate (FDR) criterion to correct for multiple testings (Benjamini and Hochberg FDR) as previously described [35, 36]. Pathway analyses were performed using Ingenuity Pathway Analysis (IPA) program (Ingenuity Systems, Redwood City, CA, USA http://www.ingenuity.com/). Differentially expressed genes were tested for statistical overrepresentation in specific canonical pathways in the Ingenuity Knowledge Base. The significance of the association between gene data set and canonical pathway was determined by the ratio of number of differential expressed genes to the total number of molecules in the specific pathway and the *P* value determined by Fisher's exact test.

### 2.3. Promoter Analysis

Prediction of AR binding sites on the promoter regions (within 1 kilobase upstream of the start codon) of genes differentially expressed between AA cancers versus CA cancers was performed using the ALGGEN-PROMO program (http://alggen.lsi.upc.es/). Maximum matrix dissimilarity rate was set at 5% as criterion to predict the conserved AR binding sites on the target genes as previously described [35].

### 2.4. PCa Cell Lines and Culture Conditions

PCa cell lines VCaP and MDA PCa 2b were purchased from American Type Culture Collection (Manassas, VA, USA). The E006AA cell line was kindly provided by Dr. Johng Rhim. VCaP and E006AA cells were maintained in DMEM medium (Life Technologies, Gaithersburg, MD) supplemented with 10% fetal bovine serum (FBS) and penicillin/streptomycin, while MDA PCa 2b cells were grown in BRFF-HPC-1 medium supplemented with 20% FBS and penicillin/streptomycin. All the cell lines were grown at 37°C and 5% CO_2_. To examine the stimulation of gene expression and cell invasion by DHT (Sigma, Kansas City, MO, USA), cells were removed from DMEM/10%FBS or BRFF-HPC1 medium and cultured in DMEM medium containing 0.1% FBS for 24 hr and subsequently treated with or without DHT (10 nM or 100 nM) for 18–24 hr. 

### 2.5. RNA Isolation and Real-Time PCR

PCa cell lines were grown to 70%–80% confluence, washed with PBS, scraped, centrifuged, and resuspended in Trizol reagent (Invitrogen, Carlsbad, CA, USA). RNAs from PCa biopsy specimens that were expression profiled by microarray analysis were also subjected to real-time RT-PCR validation assays. For PCa cell lines, RNA were isolated as described for PCa biopsy specimens (see above, Section  2.2). Two *μ*g of RNA was used for cDNA synthesis. The reverse transcription was performed with random hexamer primer using TaqMan Reverse Transcription Reagents (Applied Biosystems, Foster City, CA, USA). Real-time PCR was performed using SYBR Green PCR Master Mix from Applied Biosystems (Foster City, CA, USA). Three to five independent experiments were assayed in duplicate and normalized to levels of house-keeping genes *EIF1AX* and *PPA1* (GenBank accession numbers NM_001412 and NM_021129, resp.) [38]. We have demonstrated that *EIF1AX* and *PPA1 *are stably expressed and resistant to expression changes under different conditions and hence serve as ideal endogenous control genes for qRT-PCR assays [38]. Log2 ratio increase/decrease comparisons were calculated based on the ΔΔCt method [39]. Primer sequences are provided in Supplemental Table S1 in Supplementary Material available online at doi: http//dx.doi.org/10.1155/2013/763569. 

### 2.6. Western Blot and Chromatin Immunoprecipitation Assays

Western blotting assays were performed as previously described [35]. Rabbit polyclonal antibodies used for western blot analysis of the AR (sc-815), ITGB5 (sc-14010), and p110*β* (sc-603) were purchased from Santa Cruz Biotechnology (Santa Cruz, CA, USA). Rabbit polyclonal antibodies for RHOA (ab54835) and *β*-actin (ab8227) were purchased from Abcam (Cambridge, MA, USA). Chromatin immunoprecipitation (ChIP) Assay Kit was purchased from Millipore (Billerica, MA, USA), and ChIP assays were performed as previously described [35]. Anti-AR antibody (C-19, sc-815) used for ChIP assays was from Santa Cruz Biotechnology (Santa Cruz, CA, USA). In ChIP-PCR experiments, the quantification of AR occupancy on AR-target genes was calculated by measuring the ratios of ChIP-to-Input, and the nonantibody-treated chromatin immunoprecipitated sample served as a negative control for ChIP assays. Each quantitative PCR assay was run for 30–35 cycles. All primers used for ChIP-PCRs are listed in Supplemental Table S2. 

### 2.7. Matrigel Invasion Assay

Invasion assay was performed using BD BioCoat Matrigel Invasion Chamber (BD, San Jose, CA, USA) as previously described [35, 38]. Briefly, 750 *μ*L of growth media (with 10% FBS in DMEM or 20% FBS in BRFF-HPC1) was added into each bottom well of a 24-well plate and inserts were placed individually into each well. The E006AA cells or MDA PCa 2b cells in 200 *μ*L serum-starved media (DMEM or BRFF-HPC1 with 0.1% FBS) were seeded into each top well. After 48 hr, the invading cells were fixed, stained, and counted under light microscope as previously described [38]. Each condition was assayed in duplicate repeats from at least three independent experiments. For gene knockdown experiments, E006AA or MDA PCa 2b cells were plated at 25–35% confluence in 6-well plates one day prior to knockdown in serum-starved media (DMEM or BRFF-HPC1 with 0.1% FBS) and allowed to adhere for at least 12 hr. Chemically synthesized and purified siRNAs directed against human *RHOA, ITGB5*, and *PIK3CB* were purchased from Dharmacon (Lafayette, CO, USA), and siRNA against human *PRKD1* was purchased from Ambion (Foster City, CA, USA). Cells were transfected with 50 nmol/L siRNA using DharmaFECT 4 reagent from Thermo Fisher Scientific (Lafayette, CO, USA) for 24 hr according to the manufacturer's protocol. Scrambled nonsense siRNA sequence served as a control. After 24 hr the cells were trypsinized and seeded in a matrigel invasion assay as described above. RNA from a portion of cells was retained for RT-PCR studies to verify knockdown efficiency (typically 80% knockdown, data not shown). The target sequences of the siRNAs are as follows: *RHOA*, CGACAGCCCUGAUAGUUUA; *ITGB5*, GCUCGCAGGUCUCAACAUA; *PIK3CB*, GGAUUCAGUUGGAGUGAUU, and *PRKD1,* CGGCAAAUGUAGUGUAUUA. Experiments were conducted in duplicate repeats from at least three independent experiments.

## 3. Results

### 3.1. Overrepresentation of Differentially Expressed Genes in the AR Signaling Pathway of AA PCa Specimens

Microarray analysis revealed that 1,169 genes were differentially expressed between AA PCa and patient-matched normal prostate. By comparison, a total of 865 differentially expressed genes were identified in the analysis of CA PCa versus patient-matched normal specimens. Principal Component Analysis (PCA) demonstrated clear separation and consistency of gene expression profiles in PCa specimens versus patient-matched normal tissues in both the AA and CA populations (Supplemental Figure S1). The gene lists from these two comparisons (Supplemental Table S3) were imported into Ingenuity Pathway Analysis (IPA) to identify significant overrepresentation of differentially expressed genes residing in canonical pathways associated with PCa progression. IPA revealed 21 overrepresented canonical pathways in the cancer versus normal comparison of AA patients (*P* < 0.05), but not in the corresponding comparison involving the CA patients (*P* > 0.05, see Supplemental Table S4). These pathways included the phosphoinositide 3-kinase, protein kinase A, tumor necrosis factor receptor, and AR signaling pathways. As depicted in Supplemental Figure S2a, there were seven upregulated genes (*ARA55*, *GNAO1, GNB3, POLR2L, PRKCE, PRKD1,* and *TBP*) and one down-regulated gene (*CALR*), leading to a statistically significant (*P* < 0.05) overrepresentation in the AR signaling pathway of AA PCa patients (see figure legend in Supplemental Figure S2 for full gene names). In contrast, three upregulated genes (*GNG2, GNG11,* and *GNG12*) and two down-regulated genes (*CALM1* and *NFKB2*) were identified in the AR signaling pathway of CA PCa patients, but this did not reach statistical significance for overrepresentation (Supplemental Figure S2b). It should be noted that the differentially expressed AR signaling genes in the cancer versus normal comparison of AA patients do not overlap with the corresponding set identified in the CA patients. qRT-PCR validation of *ARA55*, *GNAO1, PRKCE, PRKD1*, *TBP, *and* CALR* in AA PCa specimens and patient-matched normal tissues demonstrated a high degree of agreement with the microarray data (Supplemental Figure S2c). There is one caveat to these microarray and qRT-PCR findings. Namely, the magnitude of differential gene expression measured in cancer versus normal samples is likely compressed (i.e., underestimated) due to the fact that the cancer biopsy specimens contain on average 50% noncancerous cells.

 Earlier biochemical and genetic studies have implicated either the AR and/or higher conversion of testosterone to DHT as culprits for PCa health disparities between the AA and CA populations [14–16]. Our genomic findings support this notion of AR signaling in cancer health disparities, and advance the hypothesis that an overall generalized augmentation of AR signaling components has occurred in AA PCa. Particularly noteworthy is the androgen receptor activator 55 (gene symbol, *ARA55;* or called *TGFB1I1*), which interacts with the AR in an androgen-dependent manner, enhancing AR transcriptional activity and ligand specificity [40]. High *ARA55* expression is correlated with shorter recurrence-free survival and poorer overall survival in CRPCa [41]. Other AR signaling component genes, identified in the comparison of AA cancer versus AA patient-matched normal (Supplemental Figure S2a), have also been functionally linked to tumor progression. For example, overexpression of protein kinase C epsilon (gene symbol, *PRKCE*) promotes conversion of androgen-dependent PCa cells to an androgen-independent phenotype [42]. An activating R234H mutation in the guanine nucleotide-binding protein G_(o)_ subunit alpha (*GNAO1*) has been implicated in breast cancer [43]. Overexpression of protein kinase D1 (*PRKD1 *or called *PKD1*) is associated with PCa progression, whereby inhibition of PRKD1 reduces migratory and invasive properties of PCa cells [44] (and see Supplemental Figure S2d). Lastly, downregulation of calreticulin (*CALR*) has been established in the PCa specimens, and overexpression of *CALR* suppresses tumor growth and metastasis [45].

### 3.2. Promoter Analysis and Pathway Mapping of AR-Target Genes

**Figure 1:**
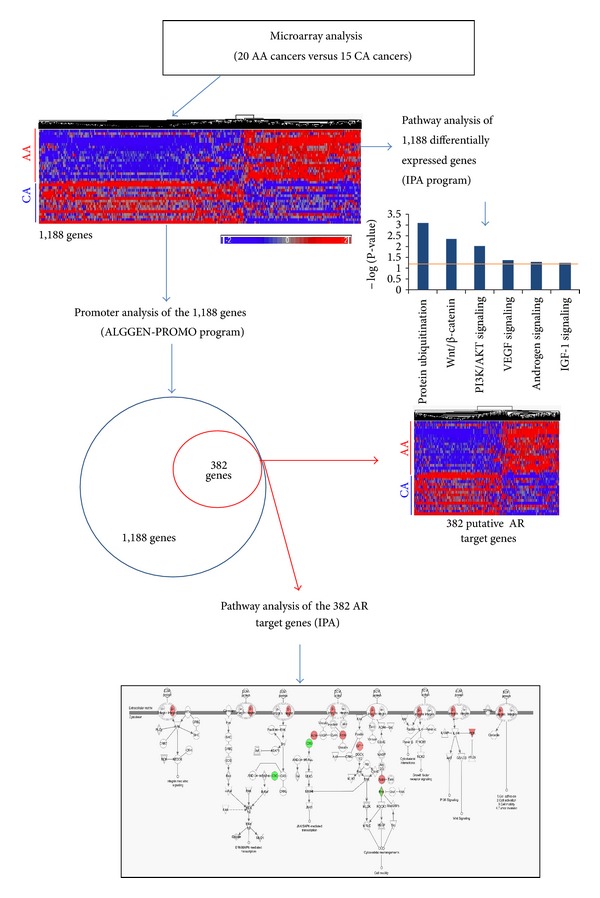
Predicted AR-target genes in AA PCa are overrepresented in cancer-associated signaling pathways. Flow chart outlining the strategy of combining gene expression profiling comparing 20 AA PCa specimens with 15 CA PCa specimens, gene promoter analysis, and pathway analysis to identify direct AR-target genes associated with PCa disparities. A total of 1,188 significant differentially express genes (ANOVA with 10% FDR) were subjected to hierarchical clustering analysis (clustering diagram, upper left, highly expressed genes in red, weakly expressed genes in blue) and pathway analysis by IPA (representative canonical pathways, upper right). Among the 1,188 significant genes, 382 genes were predicted as AR-target/responsive genes using the ALGGEN-PROMO program. These 382 genes were again subjected to clustering and pathway analysis to identify significant AR-associated pathways in AA PCa.

Based on microarray and pathway analysis results shown in Supplemental Figure S2, we hypothesized that a subset of differentially expressed genes in a comparison of AA PCa versus CA PCa would be AR-responsive/AR-target genes. Furthermore, these AR-target genes would be transcriptionally stimulated in response to the apparent increased AR signaling observed in AA PCa. To validate this hypothesis, we combined the gene expression profiling, pathway analysis, and gene promoter analysis to identify AR-target genes differentially expressed between AA and CA cancer specimens (Figure 1). First, we compared the gene profiles derived from microarray analysis of 20 AA cancers and 15 CA cancers. A total of 1,188 significant differentially expressed genes were identified in this comparison Supplemental Table S3). A Venn diagram shown in Supplemental Figure S3 depicts the number of differentially expressed genes that overlap between the AA cancer versus CA cancer comparison and our earlier 2-way comparison (AA cancer versus AA patient-matched normal and CA cancer versus CA patient-matched normal). Second, we performed IPA analysis on these 1,188 genes to identify canonical pathways with a significantly overrepresentation of differentially expressed genes. As was the case in the AA PCa versus AA patient-matched normal comparison (Supplemental Figure S2a), IPA identified the AR signaling pathway as being overrepresented in the AA PCa versus CA PCa assessment, along with a number of other signaling pathways such as the protein ubiquitination, Wnt/*β*-catenin, phosphatidylinositol-3-kinase/AKT (PI3 K/AKT), vascular endothelial growth factor (VEGF), and insulin-regulated growth factor-1 (IGF-1) signaling pathways (Figure 1). Third, we conducted a gene promoter analysis on the 1,188 significant population-associated genes in PCa to search for AR *cis*-acting binding sites. By using the ALGGEN-PROMO algorithm and a strict search criterion (5% maximal matrix dissimilarity within 1 kilobase of upstream sequence from the start of transcription), 382 out of 1,188 differentially expressed genes were identified as AR-target genes, with AR-binding sequences within 1-kb upstream of the start of transcription (Figure 1). The predicted AR-target genes and AR-binding sequences in the promoter regions are listed in Supplemental Table S5.

Lastly, we imported these 382 putative AR-target genes into IPA for further pathway analysis to identify canonical pathways overrepresented with these predicted AR-target genes (and differentially expressed between AA PCa versus CA PCa). The IPA results revealed that several cancer-associated pathways, including EGF, FGF, mTOR, integrin, JAK/STAT, and ERK/MAPK signaling pathways, were overrepresented in the AA PCa versus CA PCa comparison (Supplemental Figure S4, Supplemental Table S6). Noteworthy, many of these pathways converge onto and regulate AR signaling. Taken together, these results suggest that differential expression of AR-target genes may contribute to the more aggressive cancer phenotype (e.g., cell proliferation, antiapoptosis, invasiveness, and angiogenesis) reported in AA PCa.

### 3.3. Population-Specific PCa Cell Lines Exhibit Similar Differential Regulation of Putative AR-Target Genes as Established in PCa Specimens from AA and CA Patients

**Figure 2:**
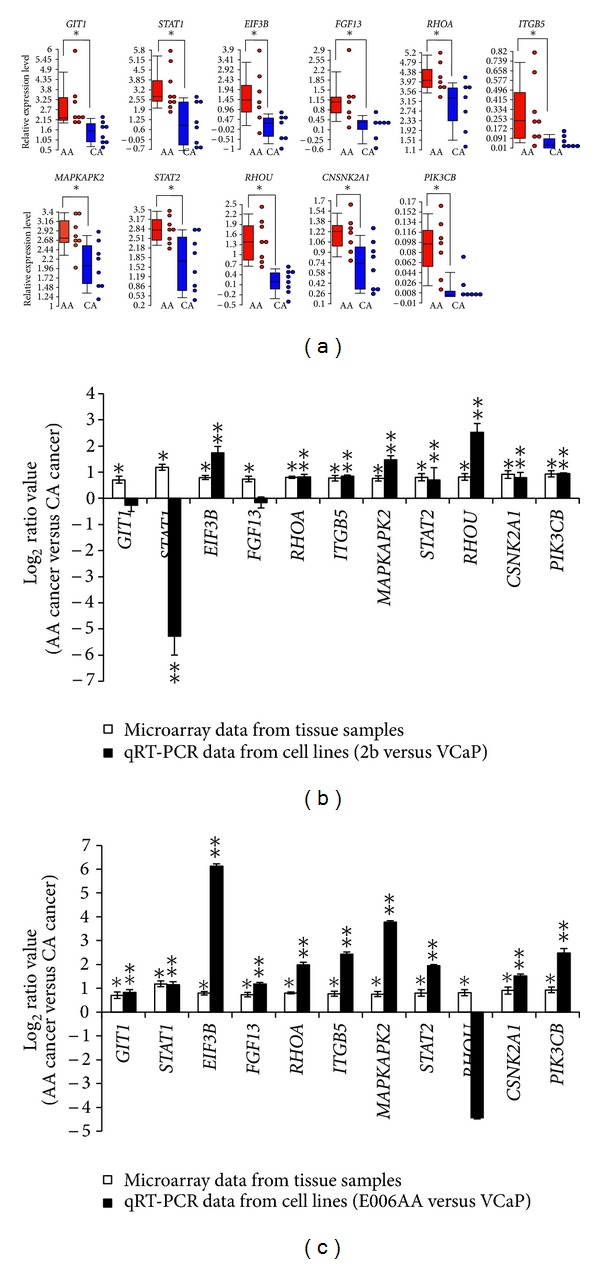
Quantitative RT-PCR validation of microarray results in patient specimens and population-specific PCa cell lines. qRT-PCR experiments were conducted to compare the gene expression levels of *GIT1*, *STAT1, EIF3B, FGF13, RHOA, ITGB5, MAPKAPK2, STAT2, RHOU, CSNK2A1, *and *PIK3CB* in (a) PCa specimens derived from AA and CA patients, (b) AA PCa cell line MDA PCa 2b (2b) versus CA PCa cell line VCaP, and (c) AA PCa cell line E006AA versus CA PCa cell line VCaP. The relative gene expression level in patient specimens (a) was determined by 2^−ΔCt^ using *EIF1AX *as the control gene for normalization. Box-and-Whisker and dot plots represent the average and individual values, respectively, of gene expression in the tested tissue samples. The log_2_ ratio values in (b) and (c) were determined by subtracting the log_2_ signal intensity of AA cancer with the log_2_ signal intensity of CA cancer for each gene from microarray results. For qRT-PCR results from cell line comparisons in (b) and (c), log⁡_2_ ratio values were calculated by the ΔΔCt method using *EIF1AX *as the control gene for normalization [35]. Data are represented as the mean ± SEM of 20 AA PCa and 15 CA PCa samples for microarray experiments and 3–5 independent PCa cell line experiments. *Significantly different between AA PCa versus CA PCa from microarray results (ANOVA, 10% FDR). **Significantly different between AA versus CA PCa cell lines from qRT-PCR results (*P* < 0.05, Student's *t*-test).

In order to further investigate the functional role of the 382 differentially expressed AR-target genes in PCa, three androgen-sensitive PCa cell lines of known racial background (VCaP, E006AA, and MDA PCa 2b) were used as *in vitro* cell models for examining AR binding, gene regulation, and associated biological functions. VCaP is a bone metastasis derived from a 59-year-old CA PCa patient [46], representing an advanced, androgen-sensitive and castration-resistant PCa cell model. E006AA was derived from a localized PCa in a 50-year=old AA patient [47], representing a primary PCa cell model. MDA PCa 2b was derived from bone metastasis from a 63-year-old AA PCa patient [48], representing an advanced and castration-resistant PCa cell model. Total RNA was purified from the PCa cell lines, and qRT-PCR was conducted to validate the expression level of 11 genes (*STAT1, STAT2, ITGB5, PIK3CB, RHOA, RHOU, FGF13, EIF3B, MAPKAPK2, GIT1,* and *CSNK2A1*). These 11 genes were originally shown to be differentially expressed in the AA cancer versus CA cancer comparison (from our microarray data) and were predicted as AR targets (see Supplemental Figure S4). We specifically selected these 11 genes for qRT-PCR validation based on their association with oncogenic pathways that were significantly overrepresented with differentially expressed genes (Supplemental Figure S4, Supplemental Table S6). As an additional validation step, the original AA PCa and CA PCa biopsy RNA (used for microarray analysis) were included for qRT-PCR analysis. qRT-PCR results indeed confirmed that all 11 genes were upregulated in the AA PCa specimens compared to CA PCa specimens (Figure 2(a)). In a comparison of gene expression levels in AA PCa cell lines versus a CA PCa cell line (i.e., MDA PCa 2b versus VCaP, or E006AA versus VCaP), there was very close agreement between qRT-PCR results in these population-specific cell lines and the microarray data in patient specimens (Figures 2(b) and 2(c)). Hence, the qRT-PCR validation results suggest that the AA cell lines (MDA PCa 2b and E006AA) and CA cell line VCaP may serve as suitable *in vitro* cell line models for investigating PCa health disparities.

### 3.4. AR Binding to Target Genes under Basal Conditions Is Higher in AA PCa Cell Lines versus CA PCa Cell Lines and AR Binding Is Increased upon Androgen Stimulation

**Figure 3:**
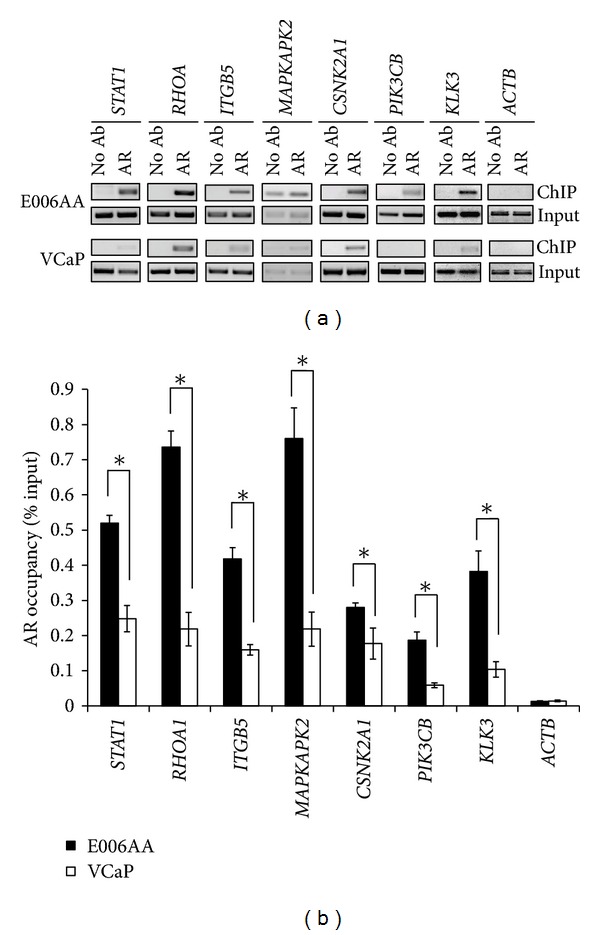
Enrichment of AR binding to target genes in AA PCa cells compared to CA PCa cells. (a) Representative ChIP-PCR assays in the AA PCa cell line E006AA and CA PCa cell line VCaP. ChIP DNA from AR-immunoprecipitates (AR), no antibody control (no Ab) or starting chromatin DNA (Input) was amplified using PCR with primers specific to predicted AR binding sites in the promoter regions of *STAT1, RHOA, ITGB5, MAPKAPK2, CSNK2A1, *and *PIK3CB *genes. *KLK3 *(*PSA* gene) and *ACTB* were used as positive and negative controls for the ChIP-PCR assays, respectively. (b) Quantification of AR enrichment on target genes in AA and CA PCa cell lines. The AR occupancies on the target genes were measured based on the percentages of ChIP-to-Input signals [% Input = (ChIP signal/Input signal)/dilution rate × 100%]. Quantification of ChIP and Input signals were calculated using the Image J program [37] from NIH. Data are represented as the mean ± SD (standard deviation) of 3–5 independent ChIP and PCR experiments. *Significantly different AR occupancies at AA versus CA target genes (*P* < 0.05 using Student's *t*-test).

The *STAT1, RHOA, ITGB5, MAPKAPK2, CSNK2A1,* and *PIK3CB* genes were shown to be basally upregulated in AA cell lines (MDA PCa 2b and E006AA) compared with the CA cell line VCaP (Figures 2(b) and 2(c)). To validate whether these upregulated genes, predicted to be AR targets, were associated with enriched AR binding to the promoter region of these genes, we performed ChIP-PCR assays in the AA cell line E006AA and CA cell line VCaP. Prior to performing ChIP-PCR, AR protein expression in the PCa cell lines was confirmed by western blot analysis (Supplemental Figure S5). The ChIP-PCR results demonstrated that AR occupancy was significantly higher for all 6 tested genes (*t*-test, *P* < 0.05) in E006AA cells compared to VCaP cells (Figures 3(a) and 3(b)), suggesting that the enriched AR binding of these 6 cancer-associated genes may be related to differential cancer aggressiveness in AA PCa versus CA PCa. As an internal control, we also demonstrate higher AR binding to the gene for prostate-specific antigen (*KLK3*), a known AR-responsive gene [49], in E006AA cells (Figures 3(a) and 3(b)). The *ACTB* gene served as a negative control for these experiments (Figures 3(a) and 3(b)).

**Figure 4:**
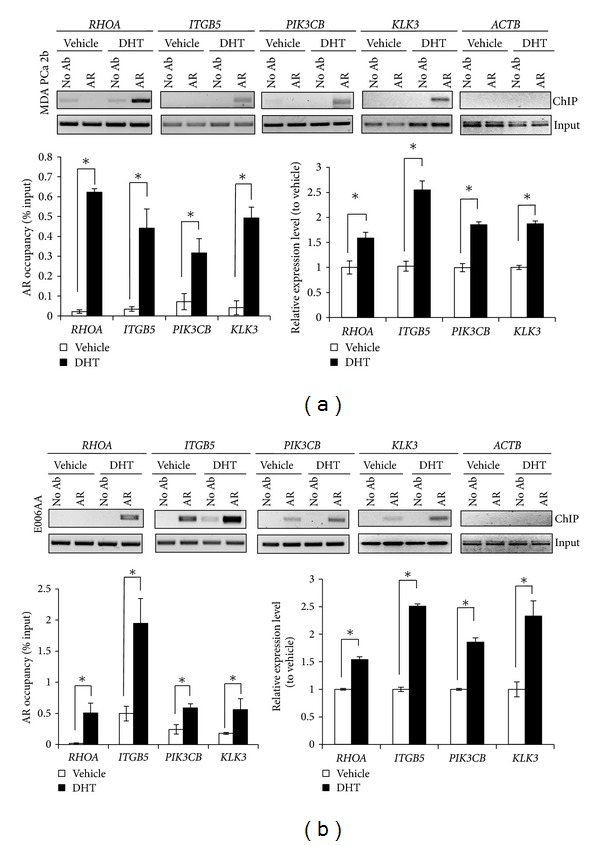
Androgen stimulation increases AR occupancy at target genes and upregulates AR-target gene expression in AA PCa cells. (a) AA PCa cell line MDA PCa 2b and (b) AA PCa cell line E006AA were treated with 100 nM DHT for 18 hr (top panels). Representative ChIP-PCR assays confirmed DHT-induced increases in AR occupancies at the promoter regions of *RHOA, ITGB5,* and *PIK3CB* genes. AR occupancies and relative gene expressions with or without androgen stimulation (bottom panels) were measured as a percentage of ChIP-to-Input signal and mRNA levels, respectively. *KLK3 *(*PSA* gene) and *ACTB* were used as positive and negative controls for the ChIP-PCR assays, respectively. For qRT-PCR, the relative expression levels of *RHOA*, *ITGB5*, *PIK3CB,* and *KLK3 *were determined by the ΔΔCt method using *EIF1AX* and *PPA1 *as endogenous genes for normalization. Cells were treated with a vehicle (<0.01% ethanol final concentration) or DHT for 18 hr prior to ChIP and qRT-PCR assays. Data are represented as the mean ± SEM of 3–5 independent ChIP and PCR experiments. *Significantly different AR occupancies and expression levels of AR-target genes in both cell lines (*P* < 0.05 using Student's *t*-test).

 DHT is a bioactive AR ligand, and its affinity to the AR is threefold greater than testosterone and 15–30-fold higher than adrenal androgens [50]. To address the effects of DHT on our predicted AR-target gene set, we next examined the occupancy of AR to the promoters of the *RHOA*, *ITGB5,* and *PIK3CB* genes in PCa cell lines. Cell lines were serum starved for 24 hr prior to stimulation with 100 nM DHT for 18 hr. ChIP-PCR results revealed DHT-stimulated increases in AR protein binding to the promoter regions of the *RHOA, ITGB5, *and *PIK3CB* genes in both AA cell lines, MDA PCa 2b (Figure 4(a)) and E006AA (Figure 4(b)). To investigate whether the enrichment of AR binding was associated with increased gene expression of the AR-target genes, qRT-PCR was performed on *RHOA, ITGB5, *and *PIK3CB *in cells serum starved for 24 hr followed by 100 nM DHT for 18 hr. *KLK3 *was included as a qRT-PCR positive control. Consistent with the ChIP-PCR results, the expression levels of *RHOA, ITGB5,* and *PIK3CB *were significantly increased in both AA cell lines in response to DHT (Figures 4(a) and 4(b), bottom right panel). Similar results were obtained when cells were serum starved for 48 hr followed by 100 nM DHT for 18 hr (Supplemental Figure S6). Moreover, comparable findings were observed with 10 nM DHT treatment for 18 hr (data not shown). It should be noted that DHT stimulation did not significantly alter AR mRNA expression in MDA PCa 2b and E006AA cells (Supplemental Figure S7). In summary, these results demonstrate that basal AR binding to target genes is higher in an AA PCa cell line compared to a CA PCa cell line, and androgen stimulation can further increase AR binding and expression of target genes in AA PCa cell lines, supporting the notion of a more aggressive PCa phenotype in AAs.

### 3.5. Activation of AR-Target Genes Contributes to the PCa Aggressiveness of AA PCa Cell Lines 

**Figure 5:**
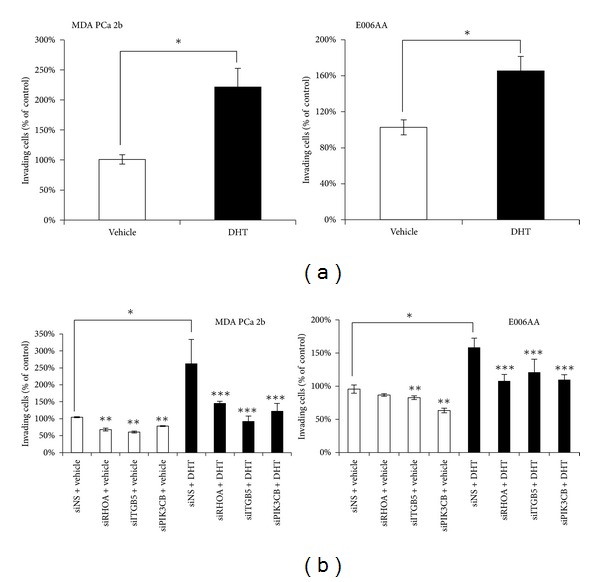
AR-target genes promote cell invasion in an androgen-dependent manner in AA cancer cells. (a) Androgen stimulation (10 nM DHT for 48 hr) increases invasion in the AA PCa cell lines MDA PCa 2b and E006AA. (b) siRNA-mediated knockdown of AR-target genes *RHOA, ITGB5,* or *PIK3CD* diminished androgen-induced (10 nM DHT for 48 hr) cell invasion in both MDA PCa 2b and E006AA cell lines. Knockdown efficiency (determined for each experiment) was typically ~80% as determined by qRT-PCR (data not shown). Data are represented as the mean ± SEM of 4–7 independent cell invasion assays. *Significant difference between cells treated with vehicle (<0.01% ethanol final concentration) versus 10 nM DHT using an ANOVA with Holm post hoc test (*P* < 0.05). **Significant difference between vehicle-treated cells incubated with siRNA against *RHOA*, *ITGB5*, or *PIK3CB* versus vehicle-treated cells incubated with nonsense siRNA (siNS) using an ANOVA with Holm post hoc test (*P* < 0.05). ***Significant difference between DHT-stimulated cells incubated with siRNA against *RHOA*, *ITGB5*, or *PIK3CB* versus DHT-stimulated cells incubated with nonsense siRNA (siNS) using an ANOVA with Holm post hoc test (*P* < 0.05).

To investigate whether the increased AR occupancy and up-regulation of AR-target genes contribute to PCa aggressiveness in AAs, the AA cell lines (MDA PCa 2b and E006AA) were seeded onto matrigel to examine their cell invasion potentials in response to DHT stimulation. After 48 hr incubation with 10 nM of DHT, a significant enhancement of invasion occurred in both MDA PCa 2b and E006AA cells (Figure 5(a)). To validate the involvement of AR-target genes in DHT-stimulated AA PCa cell invasion, the invasion potentials of MDA PCa 2b and E006AA cells were assessed following siRNA-mediated knockdown of *RHOA, ITGB5, *or *PIK3CB. *All 3 genes have been previously reported as possible invasion genes associated with breast, lung, colon, testicular, and head and neck cancers [51–55]. In PCa, RhoA has been linked to cell invasion [56–60], but has not been previously reported as a direct AR-target gene (see Figure 4) involved in androgen-stimulated PCa invasion and disparities*. ITGB5* and *PIK3CB* have not been associated with PCa invasion or disparities. There was a significant increase in invasion of nonsense siRNA- (siNS-) treated AA PCa cell lines (MDA PCa 2b and E006AA) incubated with 10 nM DHT for 48 hr compared to siNS-treated cells incubated with vehicle control (Figure 5(a)). However, this increase in DHT-stimulated invasion was abrogated with prior knockdown of *RHOA, ITGB5, *or *PIK3CB* in both AA PCa cell lines (Figure 5(b), closed bars). Interestingly, individual knockdown of *RHOA, ITGB5, *and *PIK3CB *slightly reduced the basal (i.e., vehicle treatment/no DHT) invasion potential of the AA cell lines by 20–40% (Figure 5(b), open bars), suggesting that the weak AR binding observed in some target genes under androgen-limited conditions may still contribute to PCa progression/invasion (see vehicle treatment *ITGB5* and *PI3KCB* in Figure 4(b) and [61]). All gene knockdowns were confirmed by qRT-PCR (with typical knockdowns of ~80% or more; data not shown) and western blot analysis (Supplemental Figure S8). Taken together, the results of the siRNA knockdown and matrigel invasion assays strongly suggest that up-regulation of our set of AR-target genes contributes to a more aggressive cancer phenotype (such as invasiveness) in AA cancers.

## 4. Discussion

The data resulting from our integrated approach of gene expression profiling, promoter analysis, pathway analysis, and functional validation has provided a novel genomic view of PCa disparities. This study highlights several potentially important genetic and molecular mechanisms underlying PCa disparities. We have identified a number of signaling pathways that have not been previously implicated in PCa disparities. Earlier microarray studies directly comparing AA and CA PCa specimens identified differentially expressed genes that were overrepresented in autoimmunity, inflammation, chromatin-mediated maintenance, and progesterone signaling pathways [32, 33]. Overrepresentation analysis portends potential biological significance and insight [62] into PCa disparities. Our results have revealed additional pathways, particularly cancer-associated pathways such as the AR, Wnt/*β*-catenin, PI3 K/AKT, VEGF, and IGF-1 signaling pathways (see Figure 1), which were statistically overrepresented with differentially expressed genes identified in AA PCa versus AA patient-matched normal prostate, but not in CA PCa versus CA patient-matched normal prostate comparisons. It should be noted that while the AKT and mammalian target of rapamycin (AKT/mTOR), AR, and Wnt/*β*-Catenin signaling pathways have been shown to be activated at the biochemical level (i.e., phosphorylation/activation of signaling proteins, nuclear translocation of signal proteins) in PCa specimens and cell lines, these earlier studies were not focused on PCa disparities and hence did not take into account the race/ethnicity of the patients from which the tissues were derived [63–65]. Taken together, our integrated approach has established that these cancer-associated signaling pathways are preferentially affected at the transcriptional level during PCa disparities. The direction and magnitude of the gene expression changes seen in AA PCa, but not CA specimens, suggest preferential activation of these pathways in AA PCa. 

The regulation of the AR signaling pathway in PCa is complex. Previous studies suggest that PI3 K/AKT and AR signaling pathways play complementary roles in maintaining PCa proliferation in low-androgen environments [66], and that activated PI3 K/AKT signaling can contribute to the ligand-independent and constitutive activation of the AR in CRPCa [67]. Moreover, AKT is involved in the direct phosphorylation of AR [66, 68] and potentially contributes to androgen-independent survival and growth of PCa [68]. **β**-catenin is an oncoprotein and a transcriptional coactivator of AR [69–71], and upregulated Wnt/**β**-catenin signaling has been implicated in advanced and metastatic PCa [72]. Growth factor signaling pathways (e.g., EGF, FGF, IGF-1, IGF-2, TGF*α*, and KGF) can also stimulate AR activation; the overexpression of growth factor proteins has been implicated in the transition to androgen-independent PCa [65]. Furthermore, IGF-1 overexpression has been shown to stimulate the transcription of AR-responsive genes [73]. Taken all together, the up-regulation of the PI3 K/AKT, Wnt/**β**-catenin, and IGF-1 signaling pathways may converge and contribute to the overall activation of AR signaling. Interestingly, these same pathways were observed in our study to be preferentially affected at the transcriptional level in comparisons of AA PCa versus CA PCa specimens. This strongly suggests that the AR signaling pathway may play a central role in the molecular regulation of PCa disparities. Thus, our genomic data offers a novel and unforeseen twist to the role of AR signaling in PCa disparities, beyond earlier genetic and biochemical evidence for increased levels of DHT and AR in the AA population [12–14]. We now demonstrate that an overrepresentation of gene expression changes has specifically occurred in multiple signaling pathways converging on the AR (i.e., “transcriptional convergence”). These changes have arisen preferentially or to a greater extent in AA PCa specimens compared to CA specimens. This in turn would presumably lead to transcriptional activation of downstream AR-target genes involved in oncogenesis, consequently contributing to greater PCa aggressiveness in the AA population

Given the general importance of the AR pathway in PCa progression, and the association of this pathway at the biochemical [14–16], genetic [15–19], and transcriptional levels (current study) with PCa disparities, we decided to focus on the direct downstream targets of AR. Our results have identified a number of novel AR-target genes and AR-downstream signaling pathways associated with PCa aggressiveness in AA. Previous studies have applied DNA microarray expression profiling to identify “AR-regulated genes,” defined by the differential expression of genes following vehicle and androgen treatment of the CA PCa cell lines LNCaP, VCaP, and PC3 [34, 74–76]. It remains to be determined whether these “AR-regulated genes” are direct or indirect targets of the AR. Our study is the first report to combine microarray analysis of PCa patient samples with promoter analysis and ChIP-PCR validation in order to identify direct AR-target genes associated with PCa disparities. Of the more than 1,188 genes identified in our study as being differentially regulated between AA PCa and CA PCa specimens, 382 genes were shown to have AR binding sites in their promoter region. A comparison of our 382 predicted AR-target genes with “AR-regulated genes” in CA PCa cell lines LNCaP, VCaP, and PC3 [75, 76] revealed 124 genes in common. We have also compared our AR-target gene list with ChIP-chip and ChIP-seq data derived from the CA PCa cell lines LNCaP, VCaP, and PC3 [75–77]. There were 147 AR-target genes from our dataset in common with the ChIP-chip or ChIP-seq datasets. This partial overlap is not unexpected given our criteria for identifying AR-target genes, namely that target genes must (1) be differentially expressed between AA and CA PCa biopsy specimens (likely the main reason), (2) have AR binding sites in the promoter region, and (3) have high probability for successful validation by ChIP-PCR in AA and CA PCa cell lines. The high success rate of our ChIP-PCR validation was critical in the establishment of our AR-target gene list. All 6 tested genes (*STAT1*, *RHOA*, *ITGB5*, *MAPKAPK2*, *CNSK2A1,* and *PIK3CB*), exhibiting higher expression in AA PCa specimens and AA PCa cell lines compared to the corresponding CA counterpart tissues/cell line, also displayed higher AR binding to their gene promoters in the AA PCa cell line E006AA compared to the CA PCa cell line VCaP (representing 100% success rate for validation). These 6 genes were chosen because of their overrepresentation in oncogenic pathways for EGF, FGF, mTOR, and integrin signaling (see Supplemental Figure S4), and because they were not identified in the ChIP-chip study on CA PCa cell line LNCaP [77]. Lastly, we confirmed the ability of the androgen ligand DHT to increase AR binding to the gene promoters of *RHOA*, *ITGB5,* and *PIK3CB*, which corresponded to an increase in mRNA levels in the AA PCa cell lines MDA PCa and E006AA. These findings lend further support to the validity of our AR-target gene list.

As a small GTPase, RhoA plays an important role in promoting cell invasion and migration in PCa cells [56–60]. A handful of studies have reported interactions between AR signaling and Rho-mediated signaling. It has been shown that androgen stimulation can induce RhoA activation by increasing the RhoA-GTP/total RhoA ratio in PCa cells [60, 78]. In addition, a microarray study has demonstrated that RhoA can regulate the expression of serum response factor- (SRF-) target genes in the presence of androgens [78]. However, the direct regulation of AR on *RHOA* expression and its downstream effects on PCa progression remains elusive. Our study has conclusively demonstrated for the first time that (1) *RHOA* is upregulated in both PCa specimens and cell lines derived from AA patients compared to their corresponding CA counterparts, (2) AR can directly bind to the *RHOA *promoter, (3) AR binding to the *RHOA *promoter is higher in AA compared to CA PCa cell lines, (4) DHT can further stimulate AR binding to the *RHOA *promoter in AA PCa cell lines, and (5) DHT can stimulate invasion of AA PCa cells in a *RHOA*-dependent manner.

Deregulation of integrin proteins has been associated with PCa progression. Previous studies have revealed that a number of integrin subunits, including *α*2, *α*6, *β*1, and *β*3, are overexpressed in PCa [79–82]. It has also been shown that *ITGB5 *(encoding integrin subunit *β*5) is overexpressed in prostate intraepithelial neoplasia (PIN) tissue, suggesting its potential role in the early-stage prostate tumorigenesis [83]. Moreover, cross-talk between AR signaling and protein kinase A (PKA) signaling has been implicated in PCa [74], and up-regulation of *ITGB5 *expression has been linked to PKA activation in PCa [74]. Complementary to these observations, our results have confirmed that *ITGB5 *is a direct AR-target gene, and AR-stimulated *ITGB5* mRNA up-regulation is a prerequisite for invasion by AA PCa cells.

*PIK3CB* encodes PI3K*β* (p110*β*), which is a class IA isoform of PI3 K. Accumulating evidence has suggested that PI3K*β* may play a critical role in PCa progression. Previous studies have revealed that PI3K*β* is overexpressed in PCa clinical specimens [84] and is involved in phosphorylation of AKT, promoting cell proliferation, cell survival, metabolic regulation and tumor growth in PCa [84–86]. Moreover, previous study has demonstrated that PI3K*β* is required for AR-chromatin interaction and AR-mediated gene expression [84]. However, the role of PI3K*β* in PCa disparities has not to this point been investigated. Our results have demonstrated that AR directly targets the *PIK3CB* gene in an androgen-dependent manner in AA PCa cells. More importantly, AR-stimulated *PIK3CB *expression can be linked to an increase in AA PCa cell invasion, which may explain in part PCa aggressiveness in AA patients. Of interest, KIN-193 is a drug targeting the PIK3CB protein and under investigation for the treatment of breast and prostate cancers [75].

Our list of 382 predicted AR-target genes could serve as a resource for future hypothesis-driven studies into the molecular underpinnings of PCa health disparities. Signal transducer and activator of transcription 1 (*STAT1*) represents one such example. Previous studies have demonstrated that *STAT1* is overexpressed in several human cancers [87–90]. In PCa cells, this transcriptional regulator acts as a prosurvival and chemoresistance factor [91]. These findings suggest that *STAT1* may act as a cancer driver gene; thus understanding its role in PCa health disparities would be warranted.

Cancer genome sequencing projects have revealed that mutations in hundreds of genes are involved in cancer [92–95]. Interestingly, these gene mutations tend to cluster in a limited number of pathways [92–95]. Therefore, one important lesson gleaned from the cancer genome sequencing project is that the complexity of cancer genetic alterations can be drastically reduced by focusing on these affected critical pathways. Rather than targeting multiple mutated genes at the same time, drugs are being developed to specifically target the critical pathways in cancer [96]. 

An analogous principle might be applied to the treatment of population-specific PCa disease. Multiple pathways (including the AR pathway) have an overrepresentation of differential expressed genes in comparisons of AA PCa and CA PCa specimens, and many of these overrepresented pathways converge on the AR pathway. These findings strongly suggest that AR signaling and the AR-target genes may serve as promising drug targets for the treatment of advanced PCa in the AA population. In addition, the newly discovered AR-mediated invasion genes (*RHOA, ITGB5, *and *PIK3CB*) and other AR-target genes residing in signaling pathways (such as EGF, FGF, mTOR, JAK/STAT, and ERK/MAPK signaling pathways) may extend the range of potential therapeutic options for treatment of advanced PCa in AA men.

## Supplementary Material

Supplemental Material includes Supplemental Tables S1 to S6 and Supplemental Figures S1 to S8. The Supplemental Tables are described as follows:
Supplemental Table S1: Primer sequences for qRT-PCRs, Supplemental Table S2: Primer sequences for ChIP-PCR validation of AR target genes, Supplemental Table S3: Differentially expressed genes between AA cancer versus AA matched normal, CA cancer versus CA matched normal, AA cancer versus CA cancer, and genes in common or unique in the pairwise comparisons, Supplemental Table S4: Ingenuity canonical pathways significantly over-represented in AA cancer but not in CA cancer, Supplemental Table S5: Putative AR target genes in AA PCa, Supplemental Table S6: IPA canonical signaling pathways with over-represented AR target genes in the comparison of AA PCa versus CA PCa
Supplemental Figures are described as follows:
Supplemental Figure S1: Principal component analysis (PCA) of prostate tissue specimens based on mRNA expression, Supplemental Figure S2: Over-representation of differentially expressed genes in the AR signaling pathway of AA PCa specimens, Supplemental Figure S3: Venn diagram depicting differentially expressed genes derived from pairwise comparisons of AA cancer versus AA matched normal, CA cancer versus CA matched normal, and AA cancer versus CA cancer, Supplemental Figure S4: Canonical signaling pathways with a significant over-representation of differentially expressed AR-target genes, Supplemental Figure S5: Western blot analysis of AR protein levels in CA PCa cell line VCaP and AA PCa cell lines E006AA and MDA PCa 2b, Supplemental Figure S6: DHT-stimulated gene expression after 24-hr and 48-hr serum starvation, Supplemental Figure S7: Effect of DHT treatment on AR mRNA expression in AA PCa cell lines, Supplemental Figure S8: Knockdown efficiencies of siRNAs targeting RHOA, ITGB5 and PIK3CB in PCa cells.
