# Supplementary material for: Androgen Receptor-Target Genes in African American Prostate Cancer Disparities
Source: Prostate Cancer. 2013 Jan 10;2013:763569. doi: 10.1155/2013/763569 (PMC3556896; doi:10.1155/2013/763569)
Supplement: Supplementary file 1 — Supplemental Material includes Supplemental Tables S1 to S6 and Supplemental Figures S1 to S8. The Supplemental Tables are described as follows: Supplemental Table S1: Primer sequences for qRT-PCRs, Supplemental Table S2: Primer sequences for ChIP-PCR validation of AR target genes, Supplemental Table S3: Differentially expressed genes between AA cancer versus AA matched normal, CA cancer versus CA matched normal, AA cancer versus CA cancer, and genes in common or unique in the pairwise comparisons, Supplemental Table S4: Ingenuity canonical pathways significantly over-represented in AA cancer but not in CA cancer, Supplemental Table S5: Putative AR target genes in AA PCa, Supplemental Table S6: IPA canonical signaling pathways with over-represented AR target genes in the comparison of AA PCa versus CA PCa Supplemental Figures are described as follows: Supplemental Figure S1: Principal component analysis (PCA) of prostate tissue specimens based on mRNA expression, Supplemental Figure S2: Over-representation of differentially expressed genes in the AR signaling pathway of AA PCa specimens, Supplemental Figure S3: Venn diagram depicting differentially expressed genes derived from pairwise comparisons of AA cancer versus AA matched normal, CA cancer versus CA matched normal, and AA cancer versus CA cancer, Supplemental Figure S4: Canonical signaling pathways with a significant over-representation of differentially expressed AR-target genes, Supplemental Figure S5: Western blot analysis of AR protein levels in CA PCa cell line VCaP and AA PCa cell lines E006AA and MDA PCa 2b, Supplemental Figure S6: DHT-stimulated gene expression after 24-hr and 48-hr serum starvation, Supplemental Figure S7: Effect of DHT treatment on AR mRNA expression in AA PCa cell lines, Supplemental Figure S8: Knockdown efficiencies of siRNAs targeting RHOA, ITGB5 and PIK3CB in PCa cells. [file 763569.f1.pdf]

Supplemental Figure S1

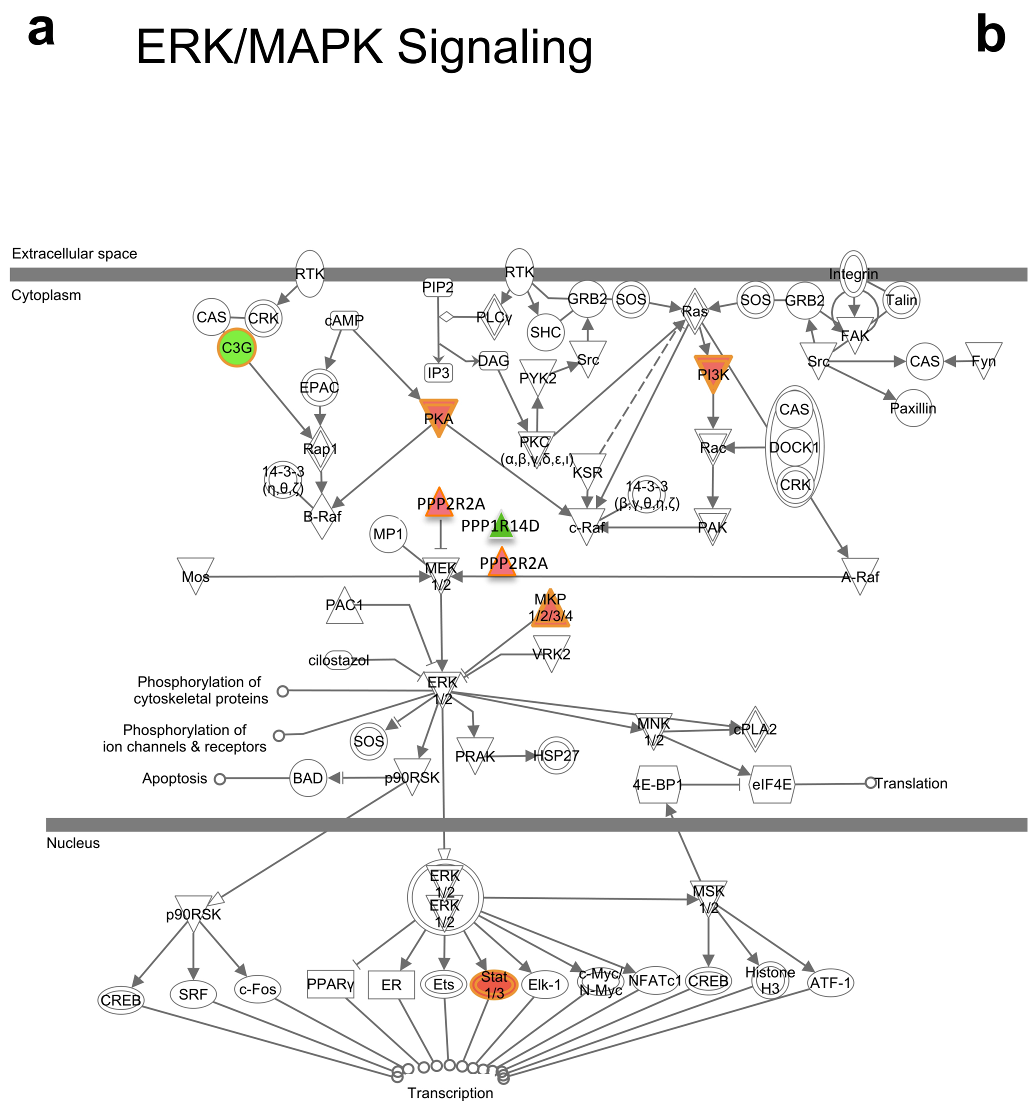

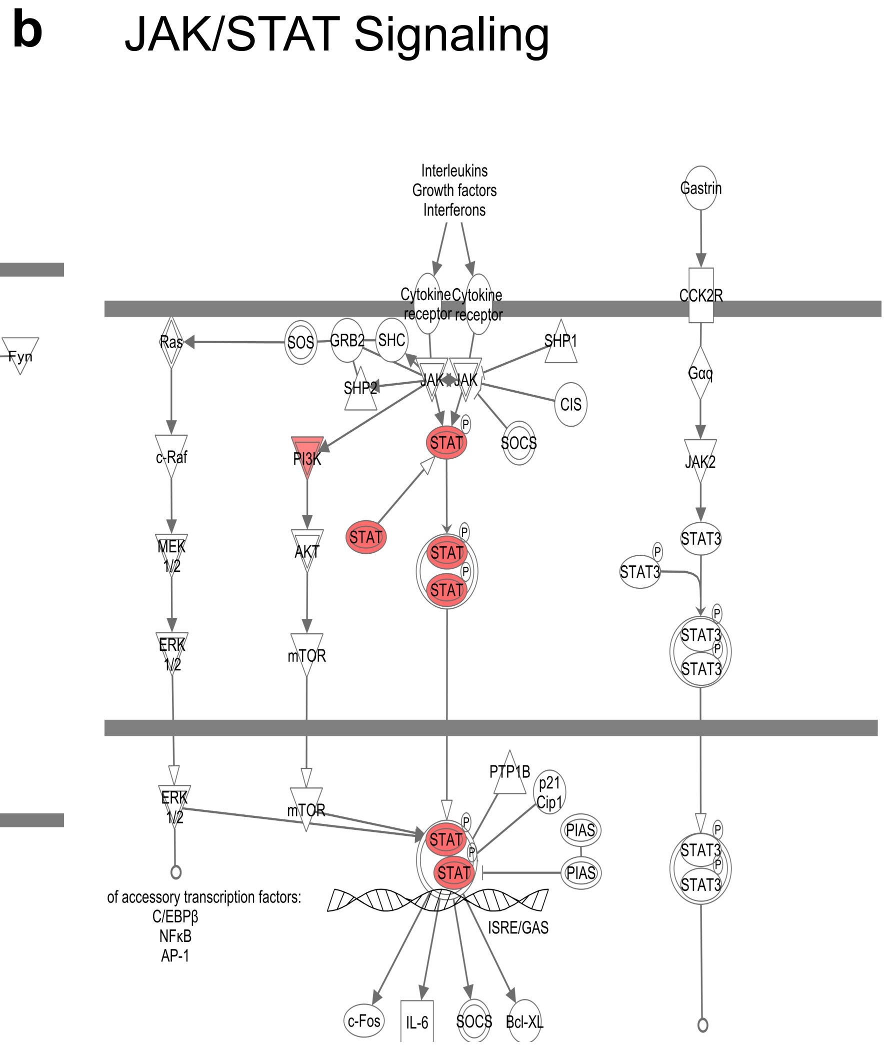

c Integrin Signaling

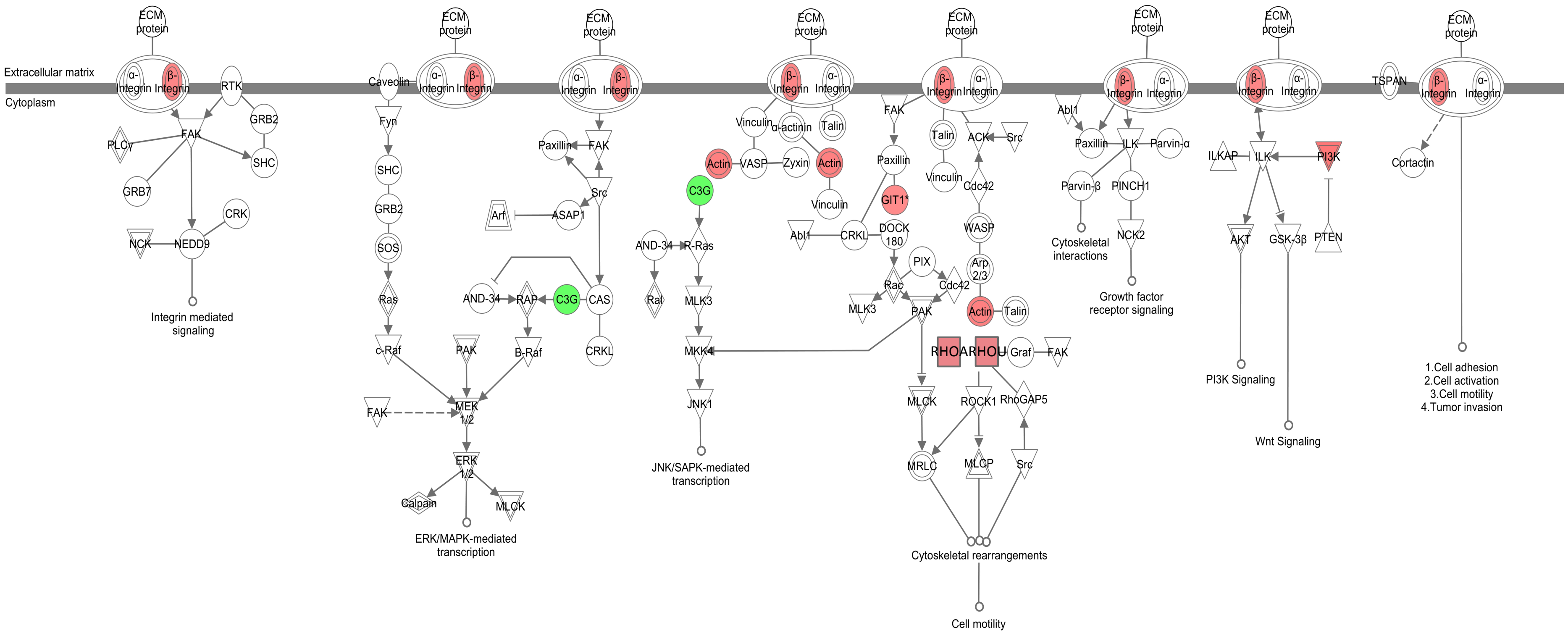

Supplemental Figure S1. Predicted AR-target genes in AA PCa are over-represented in **(a)** ERK/MAPK, **(b)** JAK/STAT and **(c)** integrin signaling pathways.

Supplemental Table S1. Primer sequenecs for qRT-PCR validation of diffentially expressed genes between AA cancer and CA cancer.

Primers	Sequences
**STAT2-F**	GAGGCCTCAACTCAGACCAG
**STAT2-R**	GCGTCCATCATTCCAGAGAT
**STAT1-F**	CCGTTTTCATGACCTCCTGT
**STAT1-R**	TGAATATTCCCCGACTGAGC
**RHOU-F**	AGGCCTCTCTGCTACACCAA
**RHOU-R**	TCAGGCACTGGCTTTTCTTT
**RHOA-F**	AAGGACCAGTTCCCAGAGGT
**RHOA-R**	TTCTGGGGTCCACTTTTCTG
**PIK3CB-F**	TCAGCCTTCGCTCCTAATGT
**PIK3CB-R**	TGCAAAGTCAGCAGGAAATG
**MAPKAPK2-F**	GAGCCCTCAGACATCTCCAG
**MAPKAPK2-R**	CCAAGAAGGGAGAAGGTTCC
**ITGB5-F**	ACAAGGGAGTCCTCTGCTCA
**ITGB5-R**	GGGGCACTTCTCACACATCT
**GIT1-F**	CCATGGACGTGTATGACGAG
**GIT1-R**	GCAAACTCTCGGGCATTAAA
**FGF13-F**	GGGTGGTATCTGGGTCTGAA
**FGF13-R**	CATTGTGGCTCATGGATTTG
**EIF3B-F**	GCCTCCTGCAGAAGAACAAC
**EIF3B-R**	CTTCCGGAAATCTTCCATCA
**CSNIK2A1-F**	ATCTTTCGGAAGGAGCCATT
**CSNIK2A1-R**	TATCGCAGCAGTTTGTCCAG

Supplemental Table S2. Primer sequences for ChIP-PCR validation of AR target genes.

AR Target Genes	Primers	Sequences
STAT1	Prom-STAT1-F	tctcacaagaggctggaggt
	Prom-STAT1-R	cagaaggaacgtgggagaag
RHOA	Prom-RHOA-F	gggattgtgcagagtggaat
	Prom-RHOA-R	catttcccttcgtggtgagt
PIK3CB	Prom-PIK3CB-F	gggcaacagtagcgaaactc
	Prom-PIK3CB-R	aaccgcgaaaaatcacagtc
MAPKAPK2	Prom-MAPKAPK2-F	tatgcagctcctttgacacg
	Prom-MAPKAPK2-R	cgtcacagcctcgtctgc
ITGB5	Prom-ITGB5-F	ttggccagtctcaaatcctt
	Prom-ITGB5-R	aaggttcctccaccttagcc
CSNK2A1	Prom-CSNK2A1-F	cccagaatgcttggctctac
	Prom-CSNK2A1-R	ccatgctgggatgtcctatt

Supplemental Table S3. Ingenuity canonical pathways that were significantly over-represented in AA cancer but not significantly over-represented in CA cancer.

Ingenuity Canonical Pathways	p-value
PI3K Signaling in B Lymphocytes	0.000630957
VDR/RXR Activation	0.002511886
Glutathione-mediated Detoxification	0.002951209
Prostanoid Biosynthesis	0.006025596
Signaling by Rho Family GTPases	0.006456542
tRNA Splicing	0.008317638
RhoGDI Signaling	0.010715193
Biotin-carboxyl Carrier Protein Assembly	0.011220185
Pyrimidine Ribonucleotides Interconversion	0.012589254
TNFR1 Signaling	0.017782794
Protein Kinase A Signaling	0.019054607
D-myo-inositol (1,4,5)-Trisphosphate Biosynthesis	0.023442288
UDP-N-acetyl-D-glucosamine Biosynthesis II	0.02630268
TNFR2 Signaling	0.033884416
TR/RXR Activation	0.034673685
Endoplasmic Reticulum Stress Pathway	0.043651583
Aldosterone Signaling in Epithelial Cells	0.043651583
Acetyl-CoA Biosynthesis III (from Citrate)	0.044668359
UDP-N-acetyl-D-galactosamine Biosynthesis II	0.046773514
Androgen Signaling	0.048621521
Lipid Antigen Presentation by CD1	0.049118723

Supplemental Table S5. IPA canonical signaling pathways with over-represented AR target genes in the comparison of AA PCa versus CA PCa

Ingenuity Canonical Pathways	p-value
Antigen Presentation Pathway	0.00025704
Glutaryl-CoA Degradation	0.000446684
Serotonin Receptor Signaling	0.00162181
Virus Entry via Endocytic Pathways	0.003019952
G-Protein Coupled Receptor Signaling	0.005011872
cAMP-mediated signaling	0.00616595
Protein Ubiquitination Pathway	0.007413102
IL-15 Production	0.008912509
tRNA Splicing	0.010964782
Fatty Acid β-oxidation I	0.010964782
Purine Nucleotides De Novo Biosynthesis II	0.012882496
Cardiac β-adrenergic Signaling	0.015488166
Glutamate Removal from Folates	0.016218101
Asparagine Biosynthesis I	0.016218101
Interferon Signaling	0.016982437
IL-12 Signaling and Production in Macrophages	0.019054607
Mechanisms of Viral Exit from Host Cells	0.025703958
JAK/Stat Signaling	0.02917427
mTOR Signaling	0.030549211
Sulfate Activation for Sulfonation	0.031622777
ERK/MAPK Signaling	0.033036954
Endoplasmic Reticulum Stress Pathway	0.033113112
Integrin Signaling	0.035237087
FGF Signaling	0.040271703
Polyamine Regulation in Colon Cancer	0.043651583
Phototransduction Pathway	0.045708819
Spermidine Biosynthesis I	0.047863009
Crosstalk between Dendritic Cells and Natural Killer Cells	0.047863009
tRNA Charging	0.047863009

Supplemental Table S4. Putative AR target genes in AA prostate cancer.

Transcript Clu	Gene Syn	gene_assignment	AR binding seq	Dissimilarity	Alignments of gene	Log2 Ratio (	Fold-Chang	Regulation (AA vs	Gene Ontology Biological Process
3735505	AANAT	NM_001088 // AAN	GGGCTGTCC	4.99562	chr17:74463650-74466198 (+) /	-0.9412	-1.9201	AA down vs CA	0007623 // circadian rhythm // inferred from electronic annotation /// 0007623
3364878	ABCC8	NM_000352 // ABC	AAAATGTCC	3.59934	chr11:17414431-17498449 (+) /	-0.80155	-1.743	AA down vs CA	0005975 // carbohydrate metabolic process // non-traceable author statement //
3364878	ABCC8	NM_000352 // ABC	AAAATGTCC	3.59934	chr11:17414492-17498358 (-) /	-0.80155	-1.743	AA down vs CA	0005975 // carbohydrate metabolic process // non-traceable author statement //
2746024	ABCE1	NM_002940 // ABC	ACACTGTCC	1.72747	chr4:146002813-146050586 (+)	1.031409	2.04402	AA up vs CA	0006401 // RNA catabolic process // traceable author statement /// 0009615 //
2746024	ABCE1	NM_002940 // ABC	TGAATGTCC	2.48409	chr4:146002813-146050586 (+)	1.031409	2.04402	AA up vs CA	0006401 // RNA catabolic process // traceable author statement /// 0009615 //
2746024	ABCE1	NM_002940 // ABC	ACACTGTCC	1.72747	chr4:146019485-146050331 (+)	1.031409	2.04402	AA up vs CA	0006401 // RNA catabolic process // traceable author statement /// 0009615 //
2746024	ABCE1	NM_002940 // ABC	TGAATGTCC	2.48409	chr4:146019485-146050331 (+)	1.031409	2.04402	AA up vs CA	0006401 // RNA catabolic process // traceable author statement /// 0009615 //
2550755	ABCG5	NM_022436 // ABC	AAATTGTCC	3.38289	chr2:44039976-44065924 (-) //	-0.81507	-1.7594	AA down vs CA	0006810 // transport // inferred from electronic annotation /// 0007584 // resp
3773932	ACTG1	NM_001614 // ACT	GGGCTGTCC	4.99562	chr1:27651826-27653034 (+) //	0.959859	1.94512	AA up vs CA	0006928 // cellular component movement // traceable author statement /// 000
3773932	ACTG1	NM_001614 // ACT	GGGCTGTCC	4.99562	chr17:79476998-79479827 (-) /	0.959859	1.94512	AA up vs CA	0006928 // cellular component movement // traceable author statement /// 000
3773932	ACTG1	NM_001614 // ACT	GGGCTGTCC	4.99562	chr17:79476996-79477482 (-) /	0.959859	1.94512	AA up vs CA	0006928 // cellular component movement // traceable author statement /// 000
3773932	ACTG1	NM_001614 // ACT	GGGCTGTCC	4.99562	chr17:79476996-79478129 (-) /	0.959859	1.94512	AA up vs CA	0006928 // cellular component movement // traceable author statement /// 000
3773932	ACTG1	NM_001614 // ACT	GGGCTGTCC	4.99562	chr17:79476996-79478284 (-) /	0.959859	1.94512	AA up vs CA	0006928 // cellular component movement // traceable author statement /// 000
3773932	ACTG1	NM_001614 // ACT	GGGCTGTCC	4.99562	chr17:79476996-79478078 (-) /	0.959859	1.94512	AA up vs CA	0006928 // cellular component movement // traceable author statement /// 000
3773932	ACTG1	NM_001614 // ACT	GGGCTGTCC	4.99562	chr17:79476996-79477559 (-) /	0.959859	1.94512	AA up vs CA	0006928 // cellular component movement // traceable author statement /// 000
3773932	ACTG1	NM_001614 // ACT	GGGCTGTCC	4.99562	chr17:79476998-79479781 (-) /	0.959859	1.94512	AA up vs CA	0006928 // cellular component movement // traceable author statement /// 000
3336801	ADRBK1	NM_001619 // ADR	GGACAGTGA	0.86373	chr11:67034157-67052936 (+) /	0.900405	1.86659	AA up vs CA	0002026 // regulation of the force of heart contraction // inferred from electronic
3336801	ADRBK1	NM_001619 // ADR	GGACAGTGA	0.86373	chr11:67034099-67054357 (+) /	0.900405	1.86659	AA up vs CA	0002026 // regulation of the force of heart contraction // inferred from electronic
3336801	ADRBK1	NM_001619 // ADR	GGACAGTGA	0.86373	chr11:67034099-67054357 (+) /	0.900405	1.86659	AA up vs CA	0002026 // regulation of the force of heart contraction // inferred from electronic
3540068	AKAP5	NM_004857 // AKA	GGAATGTCC	1.62036	chr14:64933824-64936425 (+) /	-0.8326	-1.7809	AA down vs CA	0001934 // positive regulation of protein phosphorylation // inferred from electro
3474935	ANAPC5	NM_016237 // ANA	GGTCTGTCC	4.99562	chr12:121746049-121785686 (-	0.801291	1.74266	AA up vs CA	0000086 // G2/M transition of mitotic cell cycle // traceable author statement //
3474935	ANAPC5	NM_016237 // ANA	GGTCTGTCC	4.99562	chr12:121746159-121790265 (-	0.801291	1.74266	AA up vs CA	0000086 // G2/M transition of mitotic cell cycle // traceable author statement //
3474935	ANAPC5	NM_016237 // ANA	GGTCTGTCC	4.99562	chr12:121746160-121790198 (-	0.801291	1.74266	AA up vs CA	0000086 // G2/M transition of mitotic cell cycle // traceable author statement //
3474935	ANAPC5	NM_016237 // ANA	GGTCTGTCC	4.99562	chr12:121746160-121790203 (-	0.801291	1.74266	AA up vs CA	0000086 // G2/M transition of mitotic cell cycle // traceable author statement //
2487963	ANKRD53	NM_024933 // ANK	GCCCTGTCC	4.94793	chr2:71205761-71207448 (+) //	-0.78455	-1.7226	AA down vs CA	---
2784027	ANXA5	NM_001154 // ANX	AAACTGTCC	2.73353	chr4:122589147-122618176 (-)	0.905721	1.87348	AA up vs CA	0006916 // anti-apoptosis // traceable author statement /// 0007165 // signal t
2784027	ANXA5	NM_001154 // ANX	GGACAGTCC	0.75454	chr4:122589147-122618176 (-)	0.905721	1.87348	AA up vs CA	0006916 // anti-apoptosis // traceable author statement /// 0007165 // signal t
2784027	ANXA5	NM_001154 // ANX	GCGCTGTCC	4.24108	chr4:122589147-122618176 (-)	0.905721	1.87348	AA up vs CA	0006916 // anti-apoptosis // traceable author statement /// 0007165 // signal t
3718682	AP2B1	NM_001030006 //	TTAGTGTCC	2.51915	chr17:33914292-34053428 (+) /	0.900614	1.86686	AA up vs CA	0006810 // transport // inferred from electronic annotation /// 0006886 // intra
3718682	AP2B1	NM_001030006 //	CCATTGTCC	2.8087	chr17:33914292-34053428 (+) /	0.900614	1.86686	AA up vs CA	0006810 // transport // inferred from electronic annotation /// 0006886 // intra
3718682	AP2B1	NM_001030006 //	TTAGTGTCC	2.51915	chr17:33914292-34053428 (+) /	0.900614	1.86686	AA up vs CA	0006810 // transport // inferred from electronic annotation /// 0006886 // intra
3718682	AP2B1	NM_001030006 //	CCATTGTCC	2.8087	chr17:33914292-34053428 (+) /	0.900614	1.86686	AA up vs CA	0006810 // transport // inferred from electronic annotation /// 0006886 // intra

2505833	ARHGEF4	NM_032995 // ARH	GGACAGAGC	4.24108	chr2:131674223-131804836 (+)	-0.80638	-1.7488	AA down vs CA	0006915 // apoptosis // not recorded /// 0008624 // induction of apoptosis by
2505833	ARHGEF4	NM_032995 // ARH	GGACAGAGC	4.24108	chr2:131785515-131803762 (+)	-0.80638	-1.7488	AA down vs CA	0006915 // apoptosis // not recorded /// 0008624 // induction of apoptosis by
2505833	ARHGEF4	NM_032995 // ARH	GGACAGAGC	4.24108	chr2:131675003-131804824 (-)	-0.80638	-1.7488	AA down vs CA	0006915 // apoptosis // not recorded /// 0008624 // induction of apoptosis by
3359224	ASCL2	NM_005170 // ASC	GGACAGTGA	0.86373	chr11:2290966-2291898 (-) // 9	-0.97481	-1.9654	AA down vs CA	0001666 // response to hypoxia // inferred from expression pattern /// 0001701
3359224	ASCL2	NM_005170 // ASC	GGACAGCGC	4.24108	chr11:2290966-2291898 (-) // 9	-0.97481	-1.9654	AA down vs CA	0001666 // response to hypoxia // inferred from expression pattern /// 0001701
3062576	ASNS	NM_133436 // ASN	GGACATTTT	3.59934	chr7:97481442-97501783 (-) //	0.924389	1.89788	AA up vs CA	0001889 // liver development // inferred from electronic annotation /// 000652(
2513925	B3GALT1	NM_020981 // B3G	GTATTGTCC	1.65542	chr2:168725515-168726552 (+)	-0.98036	-1.973	AA down vs CA	0006486 // protein glycosylation // inferred from direct assay /// 0006486 // pr
3921933	BACE2	NM_012105 // BAC	TAATTGTCC	2.51915	chr21:42540125-42647783 (+) /	0.927699	1.90224	AA up vs CA	0006508 // proteolysis // non-traceable author statement /// 0006508 // prote
2500275	BCL2L11	NM_138621 // BCL	GGACACCGC	4.89044	chr2:111881322-111921808 (+)	-0.78447	-1.7225	AA down vs CA	0001701 // in utero embryonic development // inferred from electronic annotatio
2500275	BCL2L11	NM_138621 // BCL	GGACACCGC	4.89044	chr2:111921737-111922208 (+)	-0.78447	-1.7225	AA down vs CA	0001701 // in utero embryonic development // inferred from electronic annotatio
3326067	C11orf41	NM_012194 // C11	GGACAGTCC	0.75454	chr11:33564473-33691683 (+) /	-0.80348	-1.7453	AA down vs CA	---
2322362	C1orf144	NM_001114600 //	GGACAGGGC	4.94793	chr1:16693725-16724640 (+) //	0.857137	1.81144	AA up vs CA	---
2322362	C1orf144	NM_001114600 //	GGACAGGGC	4.94793	chr1:16693725-16724640 (+) //	0.857137	1.81144	AA up vs CA	---
2322362	C1orf144	NM_001114600 //	GGACAGGGC	4.94793	chr1:16693725-16724640 (+) //	0.857137	1.81144	AA up vs CA	---
2322362	C1orf144	NM_001114600 //	GGACAGGGC	4.94793	chr1:16693725-16724640 (+) //	0.857137	1.81144	AA up vs CA	---
2406412	C1orf210	BC026909 // C1orf	GCCCTGTCC	4.94793	chr1:36179477-36185073 (-) //	-0.83825	-1.7879	AA down vs CA	---
2383176	C1orf95	NM_001003665 //	GAAGTGTCC	1.65542	chr1:226793429-226796915 (+)	-0.89379	-1.8581	AA down vs CA	---
2383176	C1orf95	NM_001003665 //	GCTGTGTCC	4.89044	chr1:226793429-226796915 (+)	-0.89379	-1.8581	AA down vs CA	---
2723605	C4orf19	NM_001104629 //	AGAATGTCC	3.34783	chr4:37585854-37593434 (+) //	-0.85303	-1.8063	AA down vs CA	---
2723605	C4orf19	NM_001104629 //	GGACACTAT	3.38289	chr4:37585854-37593434 (+) //	-0.85303	-1.8063	AA down vs CA	---
3095313	C8orf4	NM_020130 // C8o	GGACACTGG	2.8087	chr8:40011002-40012332 (+) //	0.96781	1.95587	AA up vs CA	0006915 // apoptosis // inferred from electronic annotation
2358092	CA14	NM_012113 // CA1	TCAGTGTCC	1.5131	chr1:150230217-150237476 (+)	-0.94157	-1.9206	AA down vs CA	---
2358092	CA14	NM_012113 // CA1	ACAGTGTCC	2.37683	chr1:150230217-150237476 (+)	-0.94157	-1.9206	AA down vs CA	---
2821194	CAST	NM_001042440 //	GGACAATTG	3.81475	chr5:96079272-96109115 (+) //	0.943644	1.92338	AA up vs CA	0007520 // myoblast fusion // inferred from electronic annotation /// 0043086 /
2821194	CAST	NM_001042440 //	GGACAATTG	3.81475	chr5:96064012-96108523 (+) //	0.943644	1.92338	AA up vs CA	0007520 // myoblast fusion // inferred from electronic annotation /// 0043086 /
2821194	CAST	NM_001042440 //	GGACAATTG	3.81475	chr5:96108879-96116208 (+) //	0.943644	1.92338	AA up vs CA	0007520 // myoblast fusion // inferred from electronic annotation /// 0043086 /
3774701	CCDC57	NM_198082 // CCD	GCTCTGTCC	4.24108	chr17:80111482-80113627 (-) /	-1.01599	-2.0223	AA down vs CA	---
3003193	CCT6A	NM_001762 // CCT	GGACACTGG	2.8087	chr7:56119455-56131682 (+) //	0.885199	1.84702	AA up vs CA	0006457 // protein folding // inferred from electronic annotation /// 0006457 //
3003193	CCT6A	NM_001762 // CCT	GGACACTGG	2.8087	chr7:56119455-56131095 (+) //	0.885199	1.84702	AA up vs CA	0006457 // protein folding // inferred from electronic annotation /// 0006457 //
2881370	CD74	NM_001025159 //	GGACATTTG	4.03121	chr5:149781299-149792313 (-)	0.90239	1.86916	AA up vs CA	0000187 // activation of MAPK activity // inferred from electronic annotation ///
3358393	CEND1	NM_016564 // CEN	GCTGTGTCC	4.89044	chr11:787116-788658 (-) // 97.	-0.80711	-1.7497	AA down vs CA	---
3737647	CHMP6	NM_024591 // CHM	GGACAGTGA	0.86373	chr17:78965702-78973931 (+) /	1.106382	2.15305	AA up vs CA	0006810 // transport // inferred from electronic annotation /// 0015031 // prot
3247712	CISD1	NM_018464 // CISD	TAAGTGTCC	2.51915	chr10:60028924-60047592 (+) /	0.896907	1.86207	AA up vs CA	0043457 // regulation of cellular respiration // inferred from electronic annotation
3029066	CLCN1	NM_000083 // CLC	GGACAATGC	0.64936	chr7:143013218-143049097 (+)	-0.78708	-1.7256	AA down vs CA	0006810 // transport // traceable author statement /// 0006810 // transport //
3029066	CLCN1	NM_000083 // CLC	GGGCTGTCC	4.99562	chr7:143013218-143049097 (+)	-0.78708	-1.7256	AA down vs CA	0006810 // transport // traceable author statement /// 0006810 // transport //
2751385	CLCN3	NM_173872 // CLC	GTAATGTCC	1.87187	chr4:170541798-170644336 (+)	0.825875	1.77261	AA up vs CA	0006810 // transport // traceable author statement /// 0006810 // transport //

2751385	CLCN3	NM_173872 // CLC	GTAATGTCC	1.87187	chr4:170541798-170644336 (+)	0.825875	1.77261	AA up vs CA	0006810 // transport // traceable author statement /// 0006810 // transport /
2751385	CLCN3	NM_173872 // CLC	GTAATGTCC	1.87187	chr4:170541798-170644336 (+)	0.825875	1.77261	AA up vs CA	0006810 // transport // traceable author statement /// 0006810 // transport /
2751385	CLCN3	NM_173872 // CLC	GTAATGTCC	1.87187	chr4:170541798-170642157 (+)	0.825875	1.77261	AA up vs CA	0006810 // transport // traceable author statement /// 0006810 // transport /
3952566	CLTCL1	NM_007098 // CLT	GAAATGTCC	1.87187	chr22:19166988-19279208 (-) /	-0.97368	-1.9639	AA down vs CA	0006886 // intracellular protein transport // inferred from electronic annotation /
2382467	CNIH3	NM_152495 // CNII	GGTCTGTCC	4.99562	chr1:224716892-224928251 (+)	-0.81477	-1.759	AA down vs CA	0023034 // intracellular signaling pathway // inferred from electronic annotation
2907459	CNPY3	NM_006586 // CNP	GGACAGTTT	2.73353	chr6:42896924-42907000 (+) /	0.804037	1.74598	AA up vs CA	0045087 // innate immune response // inferred from electronic annotation
3369755	COMMD9	NM_014186 // CON	CAAATGTCC	4.03121	chr11:36295509-36310979 (-) /	-0.83757	-1.787	AA down vs CA	---
3353417	CRTAM	NM_019604 // CRT	GGACATTTT	3.59934	chr11:122709254-122743345 (-	-0.85101	-1.8038	AA down vs CA	0001913 // T cell mediated cytotoxicity // inferred from direct assay /// 000235
2406783	CSF3R	NM_156039 // CSF	GGACAGTCT	2.48201	chr1:36931694-36948500 (-) //	-0.86537	-1.8218	AA down vs CA	0006952 // defense response // traceable author statement /// 0007155 // cell
2406783	CSF3R	NM_156039 // CSF	GGACAGTCT	2.48201	chr1:36931694-36948500 (-) //	-0.86537	-1.8218	AA down vs CA	0006952 // defense response // traceable author statement /// 0007155 // cell
2880932	CSNK1A1	NM_001025105 //	GGACAATTC	1.65542	chr5:148876302-148930651 (-)	0.931592	1.90738	AA up vs CA	0006468 // protein phosphorylation // inferred from electronic annotation /// 00
2880932	CSNK1A1	NM_001025105 //	GGACAATTC	1.65542	chr5:148872924-148885164 (-)	0.931592	1.90738	AA up vs CA	0006468 // protein phosphorylation // inferred from electronic annotation /// 00
2880932	CSNK1A1	NM_001025105 //	GGACAATTC	1.65542	chr5:148872924-148885164 (-)	0.931592	1.90738	AA up vs CA	0006468 // protein phosphorylation // inferred from electronic annotation /// 00
2880932	CSNK1A1	NM_001025105 //	GGACAATTC	1.65542	chr5:148874844-148876450 (-)	0.931592	1.90738	AA up vs CA	0006468 // protein phosphorylation // inferred from electronic annotation /// 00
2880932	CSNK1A1	NM_001025105 //	GGACAATTC	1.65542	chr5:148875555-148876308 (-)	0.931592	1.90738	AA up vs CA	0006468 // protein phosphorylation // inferred from electronic annotation /// 00
2880932	CSNK1A1	NM_001025105 //	GGACAATTC	1.65542	chr5:148875629-148876158 (-)	0.931592	1.90738	AA up vs CA	0006468 // protein phosphorylation // inferred from electronic annotation /// 00
2527786	CTDSP1	NM_021198 // CTD	GGACACTGG	2.8087	chr2:219264477-219270663 (+)	0.896357	1.86136	AA up vs CA	0006357 // regulation of transcription from RNA polymerase II promoter // inferre
2527786	CTDSP1	NM_021198 // CTD	GGACACTCG	3.56324	chr2:219264477-219270663 (+)	0.896357	1.86136	AA up vs CA	0006357 // regulation of transcription from RNA polymerase II promoter // inferre
3378344	CTSF	NM_003793 // CTS	GGACAGTCT	2.48201	chr11:66330939-66336047 (-) /	0.849087	1.80136	AA up vs CA	0006508 // proteolysis // inferred from electronic annotation /// 0006508 // pro
3634811	CTSH	NM_004390 // CTS	GGACATTTT	3.59934	chr15:79214115-79237351 (-) /	0.821465	1.7672	AA up vs CA	0006508 // proteolysis // inferred from direct assay /// 0006508 // proteolysis
3634811	CTSH	NM_004390 // CTS	GGACAGAGC	4.24108	chr15:79214115-79237351 (-) /	0.821465	1.7672	AA up vs CA	0006508 // proteolysis // inferred from direct assay /// 0006508 // proteolysis
2954355	CUL7	NM_001168370 //	GGTCTGTCC	4.99562	chr6:43005355-43021437 (-) //	0.920026	1.89215	AA up vs CA	0001570 // vasculogenesis // inferred from sequence or structural similarity /// 0
2954355	CUL7	NM_001168370 //	GGACACTGG	2.8087	chr6:43005355-43021437 (-) //	0.920026	1.89215	AA up vs CA	0001570 // vasculogenesis // inferred from sequence or structural similarity /// 0
2954355	CUL7	NM_001168370 //	GGTCTGTCC	4.99562	chr6:43005354-43021602 (-) //	0.920026	1.89215	AA up vs CA	0001570 // vasculogenesis // inferred from sequence or structural similarity /// 0
2954355	CUL7	NM_001168370 //	GGACACTGG	2.8087	chr6:43005354-43021602 (-) //	0.920026	1.89215	AA up vs CA	0001570 // vasculogenesis // inferred from sequence or structural similarity /// 0
2773958	CXCL10	NM_001565 // CXC	GGACTGTCC	0.75454	chr4:76942272-76944650 (-) //	1.074423	2.10588	AA up vs CA	0002690 // positive regulation of leukocyte chemotaxis // inferred from electroni
3272981	CYP2E1	NM_000773 // CYP	GGACAGTGG	2.15934	chr10:135340899-135352468 (-	-0.7866	-1.725	AA down vs CA	0008202 // steroid metabolic process // inferred from mutant phenotype /// 001
3272981	CYP2E1	NM_000773 // CYP	GGACAGTGG	2.15934	chr10:135340894-135352520 (-	-0.7866	-1.725	AA down vs CA	0008202 // steroid metabolic process // inferred from mutant phenotype /// 001
3272981	CYP2E1	NM_000773 // CYP	GGACAGTGG	2.15934	chr10:135340894-135352520 (-	-0.7866	-1.725	AA down vs CA	0008202 // steroid metabolic process // inferred from mutant phenotype /// 001
3272981	CYP2E1	NM_000773 // CYP	GGACAGTGG	2.15934	chr10:135340873-135351348 (-	-0.7866	-1.725	AA down vs CA	0008202 // steroid metabolic process // inferred from mutant phenotype /// 001
2400220	DDOST	NM_005216 // DDC	AAACTGTCC	2.73353	chr1:20978272-20987944 (-) //	0.804814	1.74692	AA up vs CA	0006496 // protein amino acid terminal N-glycosylation // inferred from direct ass
2400220	DDOST	NM_005216 // DDC	CAAGTGTCC	3.81475	chr1:20978272-20987944 (-) //	0.804814	1.74692	AA up vs CA	0006496 // protein amino acid terminal N-glycosylation // inferred from direct ass
2400220	DDOST	NM_005216 // DDC	AAACTGTCC	2.73353	chr1:20978680-20988000 (-) //	0.804814	1.74692	AA up vs CA	0006496 // protein amino acid terminal N-glycosylation // inferred from direct ass
2400220	DDOST	NM_005216 // DDC	CAAGTGTCC	3.81475	chr1:20978680-20988000 (-) //	0.804814	1.74692	AA up vs CA	0006496 // protein amino acid terminal N-glycosylation // inferred from direct ass
3766893	DDX5	NM_004396 // DDX	CAAGTGTCC	3.81475	chr17:62495739-62502407 (-) /	0.890229	1.85347	AA up vs CA	0006397 // mRNA processing // inferred from electronic annotation /// 0008380
3766893	DDX5	NM_004396 // DDX	CAAGTGTCC	3.81475	chr17:62495739-62502407 (-) /	0.890229	1.85347	AA up vs CA	0006397 // mRNA processing // inferred from electronic annotation /// 0008380

2427791	DENND2I	NM_024901 // DEN	ATAATGTCC	3.59934	chr1:111729802-111747031 (-)	0.973626	1.96377	AA up vs CA	---
3062523	DLX5	NM_005221 // DLX	GGACATTGT	2.59328	chr7:96649707-96654314 (-) //	-1.116	-2.1675	AA down vs CA	0001501 // skeletal system development // traceable author statement /// 0001
3062523	DLX5	NM_005221 // DLX	ACATTGTCC	2.37683	chr7:96649707-96654314 (-) //	-1.116	-2.1675	AA down vs CA	0001501 // skeletal system development // traceable author statement /// 0001
2600155	DNPEP	NM_012100 // DNF	GGACATTTA	2.73561	chr2:220238916-220252629 (-)	1.039384	2.05535	AA up vs CA	0006508 // proteolysis // inferred from electronic annotation /// 0006518 // pe
2600155	DNPEP	NM_012100 // DNF	GGACATTTA	2.73561	chr2:220249307-220252481 (-)	1.039384	2.05535	AA up vs CA	0006508 // proteolysis // inferred from electronic annotation /// 0006518 // pe
2600155	DNPEP	NM_012100 // DNF	GGACATTTA	2.73561	chr2:220238611-220252629 (-)	1.039384	2.05535	AA up vs CA	0006508 // proteolysis // inferred from electronic annotation /// 0006518 // pe
2333635	DPH2	NM_001384 // DPH	GGACATTCC	1.62036	chr1:44435699-44439041 (+) //	0.883504	1.84485	AA up vs CA	0017183 // peptidyl-diphthamide biosynthetic process from peptidyl-histidine // ir
3065601	DPY19L2	NR_027768 // DPY	TGACTGTCC	1.61828	chr12:64053558-64055463 (-) /	-1.06304	-2.0893	AA down vs CA	---
2544781	DTNB	NM_021907 // DTN	CTAATGTCC	4.03121	chr2:25600113-25610230 (-) //	0.808014	1.7508	AA up vs CA	---
2544781	DTNB	NM_021907 // DTN	CTAATGTCC	4.03121	chr2:25641562-25875529 (-) //	0.808014	1.7508	AA up vs CA	---
3464860	DUSP6	NM_001946 // DUS	GGACAAAGC	4.89044	chr12:89741838-89746164 (-) /	0.819676	1.76501	AA up vs CA	0000188 // inactivation of MAPK activity // inferred from direct assay /// 00019
3464860	DUSP6	NM_001946 // DUS	GGACAAAGC	4.89044	chr12:89741838-89746164 (-) /	0.819676	1.76501	AA up vs CA	0000188 // inactivation of MAPK activity // inferred from direct assay /// 00019
3464860	DUSP6	NM_001946 // DUS	GGACAAAGC	4.89044	chr12:89742405-89746240 (-) /	0.819676	1.76501	AA up vs CA	0000188 // inactivation of MAPK activity // inferred from direct assay /// 00019
2608801	EDEM1	NM_014674 // EDE	GAACTGTCC	1.00606	chr3:5229432-5261642 (+) // 9	0.884957	1.84671	AA up vs CA	0006986 // response to unfolded protein // inferred from electronic annotation //
2447824	EDEM3	NM_025191 // EDE	GGACAGTGC	0	chr1:184661996-184663258 (-)	0.834501	1.78324	AA up vs CA	0006986 // response to unfolded protein // inferred from electronic annotation
2447824	EDEM3	NM_025191 // EDE	GGACAGTGC	0	chr1:184659947-184723997 (-)	0.834501	1.78324	AA up vs CA	0006986 // response to unfolded protein // inferred from electronic annotation
2408643	EDN2	NM_001956 // EDN	GCTGTGTCC	4.89044	chr1:41944446-41950342 (-) //	-0.8269	-1.7739	AA down vs CA	0001516 // prostaglandin biosynthetic process // inferred from direct assay /// 0
2408643	EDN2	NM_001956 // EDN	GCGGTGTCC	4.89044	chr1:41944446-41950342 (-) //	-0.8269	-1.7739	AA down vs CA	0001516 // prostaglandin biosynthetic process // inferred from direct assay /// 0
3557408	EFS	NM_005864 // EFS	TCAGTGTCC	1.5131	chr14:23825614-23834842 (-) /	-0.86304	-1.8189	AA down vs CA	0007155 // cell adhesion // inferred from electronic annotation /// 0023034 // ir
3557408	EFS	NM_005864 // EFS	TCAGTGTCC	1.5131	chr14:23825871-23834842 (-) /	-0.86304	-1.8189	AA down vs CA	0007155 // cell adhesion // inferred from electronic annotation /// 0023034 // ir
2563654	EIF2AK3	NM_004836 // EIF2	GGACAGTCC	0.75454	chr2:88856349-88926864 (-) //	0.827201	1.77424	AA up vs CA	0001501 // skeletal system development // inferred from sequence or structural s
3591963	EIF3J	NM_003758 // EIF3	GGACAGCGC	4.24108	chr15:44829332-44854999 (+) /	0.986637	1.98156	AA up vs CA	0006412 // translation // inferred from electronic annotation /// 0006413 // tra
3591963	EIF3J	NM_003758 // EIF3	GGACAGCGC	4.24108	chr15:44829332-44854284 (+) /	0.986637	1.98156	AA up vs CA	0006412 // translation // inferred from electronic annotation /// 0006413 // tra
3362719	EIF4G2	NM_001418 // EIF4	GGACATTCC	1.62036	chr11:10818601-10830471 (-) /	0.822567	1.76855	AA up vs CA	0006412 // translation // inferred from electronic annotation /// 0006417 // reg
3362719	EIF4G2	NM_001418 // EIF4	GTAGTGTCC	1.65542	chr11:10818601-10830471 (-) /	0.822567	1.76855	AA up vs CA	0006412 // translation // inferred from electronic annotation /// 0006417 // reg
3362719	EIF4G2	NM_001418 // EIF4	GGACATTTT	3.59934	chr11:10818601-10830471 (-) /	0.822567	1.76855	AA up vs CA	0006412 // translation // inferred from electronic annotation /// 0006417 // reg
3362719	EIF4G2	NM_001418 // EIF4	GGACATTCC	1.62036	chr11:10828620-10828881 (-) /	0.822567	1.76855	AA up vs CA	0006412 // translation // inferred from electronic annotation /// 0006417 // reg
3362719	EIF4G2	NM_001418 // EIF4	GTAGTGTCC	1.65542	chr11:10828620-10828881 (-) /	0.822567	1.76855	AA up vs CA	0006412 // translation // inferred from electronic annotation /// 0006417 // reg
3362719	EIF4G2	NM_001418 // EIF4	GGACATTTT	3.59934	chr11:10828620-10828881 (-) /	0.822567	1.76855	AA up vs CA	0006412 // translation // inferred from electronic annotation /// 0006417 // reg
2495881	EIF5B	NM_015904 // EIF5	GCTGTGTCC	4.89044	chr2:99953789-100016579 (+) /	0.915796	1.88661	AA up vs CA	0006412 // translation // inferred from electronic annotation /// 0006446 // reg
2495881	EIF5B	NM_015904 // EIF5	TCATTGTCC	1.5131	chr2:99953789-100016579 (+) /	0.915796	1.88661	AA up vs CA	0006412 // translation // inferred from electronic annotation /// 0006446 // reg
2495881	EIF5B	NM_015904 // EIF5	GCTGTGTCC	4.89044	chr2:99953789-100016579 (+) /	0.915796	1.88661	AA up vs CA	0006412 // translation // inferred from electronic annotation /// 0006446 // reg
2495881	EIF5B	NM_015904 // EIF5	TCATTGTCC	1.5131	chr2:99953789-100016579 (+) /	0.915796	1.88661	AA up vs CA	0006412 // translation // inferred from electronic annotation /// 0006446 // reg
2495881	EIF5B	NM_015904 // EIF5	GCTGTGTCC	4.89044	chr2:99953899-100015997 (+) /	0.915796	1.88661	AA up vs CA	0006412 // translation // inferred from electronic annotation /// 0006446 // reg
2495881	EIF5B	NM_015904 // EIF5	TCATTGTCC	1.5131	chr2:99953899-100015997 (+) /	0.915796	1.88661	AA up vs CA	0006412 // translation // inferred from electronic annotation /// 0006446 // reg
2495881	EIF5B	NM_015904 // EIF5	GCTGTGTCC	4.89044	chr2:99980115-99980781 (+) //	0.915796	1.88661	AA up vs CA	0006412 // translation // inferred from electronic annotation /// 0006446 // reg

2495881	EIF5B	NM_015904 // EIF5	TCATTGTCC	1.5131	chr2:99980115-99980781 (+) //	0.915796	1.88661	AA up vs CA	0006412 // translation // inferred from electronic annotation /// 0006446 // reg
2495881	EIF5B	NM_015904 // EIF5	GCTGTGTCC	4.89044	chr2:99953789-100016579 (-) /	0.915796	1.88661	AA up vs CA	0006412 // translation // inferred from electronic annotation /// 0006446 // reg
2495881	EIF5B	NM_015904 // EIF5	TCATTGTCC	1.5131	chr2:99953789-100016579 (-) /	0.915796	1.88661	AA up vs CA	0006412 // translation // inferred from electronic annotation /// 0006446 // reg
2495881	EIF5B	NM_015904 // EIF5	GCTGTGTCC	4.89044	chr2:99980115-99980781 (-) //	0.915796	1.88661	AA up vs CA	0006412 // translation // inferred from electronic annotation /// 0006446 // reg
2495881	EIF5B	NM_015904 // EIF5	TCATTGTCC	1.5131	chr2:99980115-99980781 (-) //	0.915796	1.88661	AA up vs CA	0006412 // translation // inferred from electronic annotation /// 0006446 // reg
3127878	ENTPD4	NM_001128930 //	GCGGTGTCC	4.89044	chr8:23286674-23315161 (-) //	0.858474	1.81312	AA up vs CA	0006256 // UDP catabolic process // inferred from direct assay
3127878	ENTPD4	NM_001128930 //	GCGGTGTCC	4.89044	chr8:23290132-23315136 (-) //	0.858474	1.81312	AA up vs CA	0006256 // UDP catabolic process // inferred from direct assay
2677388	ERC2	NM_015576 // ERC	GGACAATGC	0.64936	chr3:55542338-56468588 (-) //	-0.80467	-1.7467	AA down vs CA	---
2677388	ERC2	NM_015576 // ERC	GCAGTGTCC	0.64936	chr3:55542338-56468588 (-) //	-0.80467	-1.7467	AA down vs CA	---
2318257	ESPN	NM_031475 // ESPI	CGAGTGTCC	3.56324	chr1:6521210-6526255 (+) // 9	-1.02459	-2.0344	AA down vs CA	0007605 // sensory perception of sound // inferred from electronic annotation //
2938196	EXOC2	NM_018303 // EXO	TGATTGTCC	2.26764	chr6:486111-619543 (-) // 99.7	0.855472	1.80935	AA up vs CA	0006810 // transport // inferred from electronic annotation /// 0006887 // exoc
2326237	EXTL1	NM_004455 // EXT	GCTCTGTCC	4.24108	chr1:26348270-26362932 (+) //	-0.87104	-1.829	AA down vs CA	0001501 // skeletal system development // traceable author statement
3838624	FCGRT	NM_001136019 //	GGACAGACC	4.99562	chr19:50016537-50029588 (+) /	0.844321	1.79542	AA up vs CA	0006955 // immune response // inferred from electronic annotation /// 0006955
2953777	FRS3	NM_006653 // FRS	GGGCTGTCC	4.99562	chr6:41737913-41745884 (-) //	-0.78044	-1.7177	AA down vs CA	0007165 // signal transduction // traceable author statement /// 0008543 // fib
2953777	FRS3	NM_006653 // FRS	GCTTTGTCC	4.89044	chr6:41737913-41745884 (-) //	-0.78044	-1.7177	AA down vs CA	0007165 // signal transduction // traceable author statement /// 0008543 // fib
3995254	GABRQ	NM_018558 // GAE	CCAATGTCC	3.02515	chrX:151806636-151821825 (+)	-1.10612	-2.1527	AA down vs CA	0006810 // transport // inferred from electronic annotation /// 0006811 // ion t
3995254	GABRQ	NM_018558 // GAE	AAATTGTCC	3.38289	chrX:151806636-151821825 (+)	-1.10612	-2.1527	AA down vs CA	0006810 // transport // inferred from electronic annotation /// 0006811 // ion t
2751936	GALNT7	NM_017423 // GAL	TTATTGTCC	2.51915	chr4:174089954-174245117 (+)	0.928063	1.90272	AA up vs CA	0005975 // carbohydrate metabolic process // traceable author statement /// 00
3794641	GALR1	NM_001480 // GAL	GGACAGCCC	4.99562	chr18:74961732-74982093 (+) /	-0.91036	-1.8795	AA down vs CA	0007165 // signal transduction // inferred from electronic annotation /// 00718
3086100	GATA4	NM_002052 // GAT	AAACTGTCC	2.73353	chr8:11565537-11617509 (+) //	-0.9491	-1.9307	AA down vs CA	0001701 // in utero embryonic development // inferred from electronic annotatio
3137875	GGH	NM_003878 // GGH	GGACATTCA	2.48409	chr8:63927640-63951386 (-) //	-0.97209	-1.9617	AA down vs CA	0006541 // glutamine metabolic process // inferred from electronic annotation //
3137875	GGH	NM_003878 // GGH	AGACTGTCC	2.48201	chr8:63927640-63951386 (-) //	-0.97209	-1.9617	AA down vs CA	0006541 // glutamine metabolic process // inferred from electronic annotation //
3969633	GLRA2	NM_002063 // GLR	GAACTGTCC	1.00606	chrX:14547795-14748718 (+) //	-0.81057	-1.7539	AA down vs CA	0006810 // transport // inferred from electronic annotation /// 0006811 // ion t
3989259	GLUD2	NM_012084 // GLU	CCATTGTCC	2.8087	chrX:120182418-120183932 (+)	0.859819	1.81481	AA up vs CA	0006520 // cellular amino acid metabolic process // inferred from electronic anno
3989259	GLUD2	NM_012084 // GLU	CCATTGTCC	2.8087	chrX:120181538-120183215 (+)	0.859819	1.81481	AA up vs CA	0006520 // cellular amino acid metabolic process // inferred from electronic anno
2622590	GNAT1	NM_144499 // GNA	CCATTGTCC	2.8087	chr3:50229052-50233949 (+) //	-0.90959	-1.8785	AA down vs CA	0007165 // signal transduction // non-traceable author statement /// 0007165 /
2622590	GNAT1	NM_144499 // GNA	CCATTGTCC	2.8087	chr3:50229035-50233948 (+) //	-0.90959	-1.8785	AA down vs CA	0007165 // signal transduction // non-traceable author statement /// 0007165 /
2624074	GNL3	NM_206825 // GNL	GGACAATTA	2.51915	chr3:52719935-52728508 (+) //	1.071927	2.10224	AA up vs CA	0008283 // cell proliferation // inferred from electronic annotation /// 0042127 /
2624074	GNL3	NM_206825 // GNL	GGACACTAC	1.65542	chr3:52719935-52728508 (+) //	1.071927	2.10224	AA up vs CA	0008283 // cell proliferation // inferred from electronic annotation /// 0042127 /
3566910	GPR135	NM_022571 // GPR	GGACATTGG	3.02515	chr14:59930509-59931996 (-) /	-0.79872	-1.7396	AA down vs CA	0007165 // signal transduction // inferred from electronic annotation /// 00718
3566910	GPR135	NM_022571 // GPR	GGACATTGG	3.02515	chr14:59930781-59931908 (-) /	-0.79872	-1.7396	AA down vs CA	0007165 // signal transduction // inferred from electronic annotation /// 00718
3566910	GPR135	NM_022571 // GPR	GGACATTGG	3.02515	chr14:59930237-59930611 (-) /	-0.79872	-1.7396	AA down vs CA	0007165 // signal transduction // inferred from electronic annotation /// 00718
2843163	GRK6	NM_001004106 //	GGACAAAGC	4.89044	chr5:176853698-176869850 (+)	1.063254	2.08964	AA up vs CA	0006468 // protein phosphorylation // inferred from electronic annotation /// 00
2843163	GRK6	NM_001004106 //	GGACAAAGC	4.89044	chr5:176853782-176869850 (+)	1.063254	2.08964	AA up vs CA	0006468 // protein phosphorylation // inferred from electronic annotation /// 00
2843163	GRK6	NM_001004106 //	GGACAAAGC	4.89044	chr5:176853843-176869624 (+)	1.063254	2.08964	AA up vs CA	0006468 // protein phosphorylation // inferred from electronic annotation /// 00
2843163	GRK6	NM_001004106 //	GGACAAAGC	4.89044	chr5:176853843-176868354 (+)	1.063254	2.08964	AA up vs CA	0006468 // protein phosphorylation // inferred from electronic annotation /// 00

2623308	GRM2	NM_000839 // GRM	GGAGTGTCC	1.4039	chr3:51742997-51752128 (+) //	-1.05319	-2.0751	AA down vs CA	0007165 // signal transduction // inferred from electronic annotation /// 00071
3011180	GRM3	NM_000840 // GRM	GGGCTGTCC	4.99562	chr7:86274076-86494189 (+) //	-0.84603	-1.7976	AA down vs CA	0007165 // signal transduction // inferred from electronic annotation /// 00071
2876479	H2AFY	NM_004893 // H2A	GGACATTGT	2.59328	chr5:134670072-134735577 (-)	0.817238	1.76203	AA up vs CA	0006334 // nucleosome assembly // non-traceable author statement /// 000633
2876479	H2AFY	NM_004893 // H2A	GGACATTGT	2.59328	chr5:134670617-134734881 (-)	0.817238	1.76203	AA up vs CA	0006334 // nucleosome assembly // non-traceable author statement /// 000633
2876479	H2AFY	NM_004893 // H2A	GGACATTGT	2.59328	chr5:134670617-134734881 (-)	0.817238	1.76203	AA up vs CA	0006334 // nucleosome assembly // non-traceable author statement /// 000633
2738723	HADH	NM_001184705 //	TCACTGTCC	0.86373	chr4:108911061-108955646 (+)	0.859421	1.81431	AA up vs CA	0006629 // lipid metabolic process // inferred from electronic annotation /// 000
2738723	HADH	NM_001184705 //	TCACTGTCC	0.86373	chr4:108911001-108956323 (+)	0.859421	1.81431	AA up vs CA	0006629 // lipid metabolic process // inferred from electronic annotation /// 000
2738723	HADH	NM_001184705 //	TCACTGTCC	0.86373	chr4:108910869-108956017 (+)	0.859421	1.81431	AA up vs CA	0006629 // lipid metabolic process // inferred from electronic annotation /// 000
2545092	HADHA	NM_000182 // HAD	CTACTGTCC	3.16539	chr12:8786930-8790876 (+) //	0.862471	1.81815	AA up vs CA	0006629 // lipid metabolic process // inferred from electronic annotation /// 000
2545092	HADHA	NM_000182 // HAD	TCACTGTCC	0.86373	chr12:8786930-8790876 (+) //	0.862471	1.81815	AA up vs CA	0006629 // lipid metabolic process // inferred from electronic annotation /// 000
2545092	HADHA	NM_000182 // HAD	CTACTGTCC	3.16539	chr12:8786930-8790876 (+) //	0.862471	1.81815	AA up vs CA	0006629 // lipid metabolic process // inferred from electronic annotation /// 000
2545092	HADHA	NM_000182 // HAD	TCACTGTCC	0.86373	chr12:8786930-8790876 (+) //	0.862471	1.81815	AA up vs CA	0006629 // lipid metabolic process // inferred from electronic annotation /// 000
2545092	HADHA	NM_000182 // HAD	CTACTGTCC	3.16539	chr12:8786936-8790367 (+) //	0.862471	1.81815	AA up vs CA	0006629 // lipid metabolic process // inferred from electronic annotation /// 000
2545092	HADHA	NM_000182 // HAD	TCACTGTCC	0.86373	chr12:8786936-8790367 (+) //	0.862471	1.81815	AA up vs CA	0006629 // lipid metabolic process // inferred from electronic annotation /// 000
2438531	HDGF	NM_004494 // HDG	GGACACAGC	4.89044	chr1:156711901-156721536 (-)	0.878419	1.83836	AA up vs CA	0006350 // transcription // inferred from electronic annotation /// 0007165 // s
2438531	HDGF	NM_004494 // HDG	GGACACAGC	4.89044	chr1:156711901-156712429 (-)	0.878419	1.83836	AA up vs CA	0006350 // transcription // inferred from electronic annotation /// 0007165 // s
2899110	HFE	NM_000410 // HFE	GGACACTGG	2.8087	chr6:26087657-26094584 (+) //	-0.80518	-1.7474	AA down vs CA	0002474 // antigen processing and presentation of peptide antigen via MHC class
2899110	HFE	NM_000410 // HFE	GGACACTGG	2.8087	chr6:26087447-26097056 (+) //	-0.80518	-1.7474	AA down vs CA	0002474 // antigen processing and presentation of peptide antigen via MHC class
2899110	HFE	NM_000410 // HFE	GGACACTGG	2.8087	chr6:26091323-26096817 (+) //	-0.80518	-1.7474	AA down vs CA	0002474 // antigen processing and presentation of peptide antigen via MHC class
2899110	HFE	NM_000410 // HFE	GGACACTGG	2.8087	chr6:26087625-26094485 (+) //	-0.80518	-1.7474	AA down vs CA	0002474 // antigen processing and presentation of peptide antigen via MHC class
2899110	HFE	NM_000410 // HFE	GGACACTGG	2.8087	chr6:26087624-26094485 (+) //	-0.80518	-1.7474	AA down vs CA	0002474 // antigen processing and presentation of peptide antigen via MHC class
2899110	HFE	NM_000410 // HFE	GGACACTGG	2.8087	chr6:26087545-26096817 (+) //	-0.80518	-1.7474	AA down vs CA	0002474 // antigen processing and presentation of peptide antigen via MHC class
2899110	HFE	NM_000410 // HFE	GGACACTGG	2.8087	chr6:26087662-26097056 (+) //	-0.80518	-1.7474	AA down vs CA	0002474 // antigen processing and presentation of peptide antigen via MHC class
2899110	HFE	NM_000410 // HFE	GGACACTGG	2.8087	chr6:26087545-26095445 (+) //	-0.80518	-1.7474	AA down vs CA	0002474 // antigen processing and presentation of peptide antigen via MHC class
2899110	HFE	NM_000410 // HFE	GGACACTGG	2.8087	chr6:26087545-26096818 (+) //	-0.80518	-1.7474	AA down vs CA	0002474 // antigen processing and presentation of peptide antigen via MHC class
2899110	HFE	NM_000410 // HFE	GGACACTGG	2.8087	chr6:26091321-26096817 (+) //	-0.80518	-1.7474	AA down vs CA	0002474 // antigen processing and presentation of peptide antigen via MHC class
2899110	HFE	NM_000410 // HFE	GGACACTGG	2.8087	chr6:26087632-26094458 (+) //	-0.80518	-1.7474	AA down vs CA	0002474 // antigen processing and presentation of peptide antigen via MHC class
2899110	HFE	NM_000410 // HFE	GGACACTGG	2.8087	chr6:26087632-26094458 (+) //	-0.80518	-1.7474	AA down vs CA	0002474 // antigen processing and presentation of peptide antigen via MHC class
2899110	HFE	NM_000410 // HFE	GGACACTGG	2.8087	chr6:26087632-26095058 (+) //	-0.80518	-1.7474	AA down vs CA	0002474 // antigen processing and presentation of peptide antigen via MHC class
2899261	HIST1H2I	NM_003525 // HIST	GCCCTGTCC	4.94793	chr6:26273203-26273584 (+) //	-0.8445	-1.7957	AA down vs CA	0006334 // nucleosome assembly // inferred from electronic annotation /// 0006
2899261	HIST1H2I	NM_003525 // HIST	GGACACCGC	4.89044	chr6:26273203-26273584 (+) //	-0.8445	-1.7957	AA down vs CA	0006334 // nucleosome assembly // inferred from electronic annotation /// 0006
3250237	HKDC1	NM_025130 // HKD	GGACAATGT	2.37683	chr10:71000405-71027305 (+) /	-0.85613	-1.8102	AA down vs CA	0005975 // carbohydrate metabolic process // inferred from electronic annotation
2948926	HLA-B	NM_005514 // HLA	CCAGTGTCC	2.8087	chr6:29795626-29913064 (+) //	0.808649	1.75157	AA up vs CA	0002223 // stimulatory C-type lectin receptor signaling pathway // inferred from
2948926	HLA-B	NM_005514 // HLA	CCAGTGTCC	2.8087	chr6:29795626-29913064 (+) //	0.808649	1.75157	AA up vs CA	0002223 // stimulatory C-type lectin receptor signaling pathway // inferred from
2948926	HLA-B	NM_005514 // HLA	CCAGTGTCC	2.8087	chr6:31321896-31325022 (-) //	0.808649	1.75157	AA up vs CA	0002223 // stimulatory C-type lectin receptor signaling pathway // inferred from
2948926	HLA-B	NM_005514 // HLA	CCAGTGTCC	2.8087	chr6:31321896-31325022 (-) //	0.808649	1.75157	AA up vs CA	0002223 // stimulatory C-type lectin receptor signaling pathway // inferred from

2948926	HLA-B	NM_005514 // HLA	CCAGTGTCC	2.8087	chr6:31322409-31324935 (-) //	0.808649	1.75157	AA up vs CA	0002223 // stimulatory C-type lectin receptor signaling pathway // inferred from
2948926	HLA-B	NM_005514 // HLA	CCAGTGTCC	2.8087	chr6:31322409-31324935 (-) //	0.808649	1.75157	AA up vs CA	0002223 // stimulatory C-type lectin receptor signaling pathway // inferred from
2901620	HLA-E	NM_005516 // HLA	GGACAGAGC	4.24108	chr6:30457288-30461970 (+) //	0.84	1.79005	AA up vs CA	0002474 // antigen processing and presentation of peptide antigen via MHC class
2901620	HLA-E	NM_005516 // HLA	GGACAGAGC	4.24108	chr6:30457288-30461970 (+) //	0.84	1.79005	AA up vs CA	0002474 // antigen processing and presentation of peptide antigen via MHC class
2901620	HLA-E	NM_005516 // HLA	GGACAGAGC	4.24108	chr6:30457302-30461055 (+) //	0.84	1.79005	AA up vs CA	0002474 // antigen processing and presentation of peptide antigen via MHC class
2901620	HLA-E	NM_005516 // HLA	GGACAGAGC	4.24108	chr6:30457302-30461055 (+) //	0.84	1.79005	AA up vs CA	0002474 // antigen processing and presentation of peptide antigen via MHC class
2901620	HLA-E	NM_005516 // HLA	GGACAGAGC	4.24108	chr6:30457336-30460991 (+) //	0.84	1.79005	AA up vs CA	0002474 // antigen processing and presentation of peptide antigen via MHC class
2901620	HLA-E	NM_005516 // HLA	GGACAGAGC	4.24108	chr6:30457336-30460991 (+) //	0.84	1.79005	AA up vs CA	0002474 // antigen processing and presentation of peptide antigen via MHC class
2401275	HNRNPR	NM_001102398 //	ATAGTGTCC	3.38289	chr1:23636015-23670801 (-) //	0.830685	1.77853	AA up vs CA	0006397 // mRNA processing // inferred from electronic annotation /// 0006397
2401275	HNRNPR	NM_001102398 //	ATAGTGTCC	3.38289	chr1:23636276-23670803 (-) //	0.830685	1.77853	AA up vs CA	0006397 // mRNA processing // inferred from electronic annotation /// 0006397
3042756	HOXA2	NM_006735 // HOX	GGACACTGG	2.8087	chr7:27139973-27142394 (-) //	-1.00312	-2.0043	AA down vs CA	0000122 // negative regulation of transcription from RNA polymerase II promoter
2825733	HSD17B4	NM_000414 // HSD	TAAATGTCC	2.73561	chr5:118788222-118878024 (+)	0.931887	1.90777	AA up vs CA	0000038 // very long-chain fatty acid metabolic process // inferred from electron
3540136	HSPA2	NM_021979 // HSP	GCTGTGTCC	4.89044	chr14:64590735-65009952 (+)	-0.82678	-1.7737	AA down vs CA	0006950 // response to stress // inferred from electronic annotation /// 000698
3540136	HSPA2	NM_021979 // HSP	GGACAGTAC	1.00606	chr14:64590735-65009952 (+)	-0.82678	-1.7737	AA down vs CA	0006950 // response to stress // inferred from electronic annotation /// 000698
2859387	HTR1A	NM_000524 // HTR	GAACTGTCC	1.00606	chr5:63256277-63257546 (-) //	-0.83711	-1.7865	AA down vs CA	0007165 // signal transduction // inferred from electronic annotation /// 000718
3513147	HTR2A	NM_000621 // HTR	GGACACTCA	2.26764	chr13:47407512-47470173 (-) /	-0.78366	-1.7215	AA down vs CA	0001659 // temperature homeostasis // inferred from electronic annotation /// 0
3513147	HTR2A	NM_000621 // HTR	GGACACTCA	2.26764	chr13:47408995-47470017 (-) /	-0.78366	-1.7215	AA down vs CA	0001659 // temperature homeostasis // inferred from electronic annotation /// 0
3349660	HTR3B	NM_006028 // HTR	TTAGTGTCC	2.51915	chr11:113775588-113816966 (-	-0.79068	-1.7299	AA down vs CA	0006810 // transport // traceable author statement /// 0006810 // transport //
3824874	IFI30	NM_006332 // IFI3	CCACTGTCC	2.15934	chr19:18284624-18288923 (+)	0.81741	1.76224	AA up vs CA	0019886 // antigen processing and presentation of exogenous peptide antigen via
3824874	IFI30	NM_006332 // IFI3	GGACACTCA	2.26764	chr19:18284624-18288923 (+)	0.81741	1.76224	AA up vs CA	0019886 // antigen processing and presentation of exogenous peptide antigen via
2772566	IGJ	NM_144646 // IGJ	GGACATTTG	4.03121	chr4:71521258-71532344 (-) //	0.805111	1.74728	AA up vs CA	0006955 // immune response // non-traceable author statement
2784279	IL21	NM_021803 // IL21	GGACATTAG	4.03121	chr4:123533782-123542212 (-)	-0.82649	-1.7734	AA down vs CA	0006955 // immune response // inferred from electronic annotation /// 0007165
2377035	IL24	NM_006850 // IL2	CTACTGTCC	3.16539	chr1:207070788-207077209 (+)	-0.79256	-1.7322	AA down vs CA	0006915 // apoptosis // inferred from electronic annotation /// 0006915 // apo
2377035	IL24	NM_006850 // IL2	GTATTGTCC	1.65542	chr1:207070788-207077209 (+)	-0.79256	-1.7322	AA down vs CA	0006915 // apoptosis // inferred from electronic annotation /// 0006915 // apo
3820663	ILF3	NM_012218 // ILF3	GGACAAAGC	4.89044	chr19:10764973-10796441 (+)	0.79263	1.73223	AA up vs CA	0000279 // M phase // non-traceable author statement /// 0006350 // transcrip
3820663	ILF3	NM_012218 // ILF3	GGACAAAGC	4.89044	chr19:10781269-10796150 (+)	0.79263	1.73223	AA up vs CA	0000279 // M phase // non-traceable author statement /// 0006350 // transcrip
3820663	ILF3	NM_012218 // ILF3	GGACAAAGC	4.89044	chr19:10781189-10800442 (+)	0.79263	1.73223	AA up vs CA	0000279 // M phase // non-traceable author statement /// 0006350 // transcrip
3820663	ILF3	NM_012218 // ILF3	GGACAAAGC	4.89044	chr19:10801396-10803093 (+)	0.79263	1.73223	AA up vs CA	0000279 // M phase // non-traceable author statement /// 0006350 // transcrip
3820663	ILF3	NM_012218 // ILF3	GGACAAAGC	4.89044	chr19:10764987-10800523 (+)	0.79263	1.73223	AA up vs CA	0000279 // M phase // non-traceable author statement /// 0006350 // transcrip
3976120	INE1	NR_024616 // INE1	GGACAGTGT	1.72747	chrX:47064319-47065252 (+) //	-0.98082	-1.9736	AA down vs CA	---
2374872	IPO9	NM_018085 // IPO	TAAATGTCC	2.73561	chr1:201851913-201853419 (+)	0.856045	1.81007	AA up vs CA	0006606 // protein import into nucleus // non-traceable author statement /// 00
2374872	IPO9	NM_018085 // IPO	TAAATGTCC	2.73561	chr1:201836010-201845854 (+)	0.856045	1.81007	AA up vs CA	0006606 // protein import into nucleus // non-traceable author statement /// 00
2875348	IRF1	NM_002198 // IRF1	GGGCTGTCC	4.99562	chr5:131818779-131826427 (-)	1.043869	2.06175	AA up vs CA	0006350 // transcription // inferred from electronic annotation /// 0006355 // r
2692816	ITGB5	NM_002213 // ITGE	AAATTGTCC	3.38289	chr3:124482009-124482116 (+)	0.908467	1.87705	AA up vs CA	0006936 // muscle contraction // not recorded /// 0007155 // cell adhesion //
2692816	ITGB5	NM_002213 // ITGE	GGACAGTTT	2.73353	chr3:124482009-124482116 (+)	0.908467	1.87705	AA up vs CA	0006936 // muscle contraction // not recorded /// 0007155 // cell adhesion //
2692816	ITGB5	NM_002213 // ITGE	AAATTGTCC	3.38289	chr3:124480761-124606152 (-)	0.908467	1.87705	AA up vs CA	0006936 // muscle contraction // not recorded /// 0007155 // cell adhesion //

2692816	ITGB5	NM_002213 // ITGE	GGACAGTTT	2.73353	chr3:124480761-124606152 (-)	0.908467	1.87705	AA up vs CA	0006936 // muscle contraction // not recorded /// 0007155 // cell adhesion //
2692816	ITGB5	NM_002213 // ITGE	AAATTGTCC	3.38289	chr3:124481795-124605876 (-)	0.908467	1.87705	AA up vs CA	0006936 // muscle contraction // not recorded /// 0007155 // cell adhesion //
2692816	ITGB5	NM_002213 // ITGE	GGACAGTTT	2.73353	chr3:124481795-124605876 (-)	0.908467	1.87705	AA up vs CA	0006936 // muscle contraction // not recorded /// 0007155 // cell adhesion //
2692816	ITGB5	NM_002213 // ITGE	AAATTGTCC	3.38289	chr3:124482009-124482116 (-)	0.908467	1.87705	AA up vs CA	0006936 // muscle contraction // not recorded /// 0007155 // cell adhesion //
2692816	ITGB5	NM_002213 // ITGE	GGACAGTTT	2.73353	chr3:124482009-124482116 (-)	0.908467	1.87705	AA up vs CA	0006936 // muscle contraction // not recorded /// 0007155 // cell adhesion //
2448073	IVNS1AB	NM_006469 // IVNS	CTAATGTCC	4.03121	chr1:185265529-185286386 (-)	0.857885	1.81238	AA up vs CA	0006383 // transcription from RNA polymerase III promoter // traceable author st
2448073	IVNS1AB	NM_006469 // IVNS	CTAATGTCC	4.03121	chr1:185266043-185286468 (-)	0.857885	1.81238	AA up vs CA	0006383 // transcription from RNA polymerase III promoter // traceable author st
2448073	IVNS1AB	NM_006469 // IVNS	CTAATGTCC	4.03121	chr1:185266897-185286375 (-)	0.857885	1.81238	AA up vs CA	0006383 // transcription from RNA polymerase III promoter // traceable author st
3771773	JMJD6	NM_001081461 //	GGACAGCCC	4.99562	chr17:74714532-74722754 (-) /	-1.10235	-2.147	AA down vs CA	0001568 // blood vessel development // inferred from electronic annotation /// 0
3771773	JMJD6	NM_001081461 //	GGACAGCCC	4.99562	chr17:74714532-74722754 (-) /	-1.10235	-2.147	AA down vs CA	0001568 // blood vessel development // inferred from electronic annotation /// 0
3771773	JMJD6	NM_001081461 //	GGACAGCCC	4.99562	chr17:74708911-74722563 (-) /	-1.10235	-2.147	AA down vs CA	0001568 // blood vessel development // inferred from electronic annotation /// 0
3079202	KCNH2	NM_000238 // KCN	AGAATGTCC	3.34783	chr7:150642049-150675178 (-)	-0.79641	-1.7368	AA down vs CA	0000160 // two-component signal transduction system (phosphorelay) // inferred
3079202	KCNH2	NM_000238 // KCN	AGAATGTCC	3.34783	chr7:150646521-150675178 (-)	-0.79641	-1.7368	AA down vs CA	0000160 // two-component signal transduction system (phosphorelay) // inferred
2552051	KCNK12	NM_022055 // KCN	GCGCTGTCC	4.24108	chr2:47747916-47797470 (-) //	-0.79372	-1.7335	AA down vs CA	0006493 // protein O-linked glycosylation // inferred from electronic annotation /
2332091	KCNQ4	NM_004700 // KCN	GGACACTGC	0.64936	chr1:41249683-41304360 (+) //	-0.80203	-1.7436	AA down vs CA	0006810 // transport // inferred from electronic annotation /// 0006811 // ion t
2328465	KHDRBS1	NM_006559 // KHD	CGACTGTCC	2.91388	chr1:32479490-32509472 (+) //	0.946357	1.927	AA up vs CA	0000086 // G2/M transition of mitotic cell cycle // inferred from sequence or stru
2328465	KHDRBS1	NM_006559 // KHD	CGACTGTCC	2.91388	chr1:32479482-32508536 (+) //	0.946357	1.927	AA up vs CA	0000086 // G2/M transition of mitotic cell cycle // inferred from sequence or stru
2328465	KHDRBS1	NM_006559 // KHD	CGACTGTCC	2.91388	chr1:32479596-32526451 (+) //	0.946357	1.927	AA up vs CA	0000086 // G2/M transition of mitotic cell cycle // inferred from sequence or stru
3484895	KL	NM_004795 // KL /	GGACACTGT	2.37683	chr13:33590570-33640279 (+) /	-1.03819	-2.0537	AA down vs CA	0002526 // acute inflammatory response // inferred from electronic annotation //
3484895	KL	NM_004795 // KL /	GCAATGTCC	0.86582	chr13:33590570-33640279 (+) /	-1.03819	-2.0537	AA down vs CA	0002526 // acute inflammatory response // inferred from electronic annotation //
2452440	KLHDC8A	NM_018203 // KLH	GGACAGCCC	4.99562	chr1:205305647-205326039 (-)	-0.89639	-1.8614	AA down vs CA	---
3637367	KLHL25	NM_022480 // KLH	GGACATTTA	2.73561	chr15:86302560-86338100 (-) /	-0.83934	-1.7892	AA down vs CA	---
2877028	KLHL3	NM_017415 // KLH	TAATTGTCC	2.51915	chr5:136953188-137071463 (-)	-0.84169	-1.7921	AA down vs CA	---
3404496	KLRF1	NM_016523 // KLR	GGACAGTTG	3.16539	chr12:9980076-9997601 (+) //	-0.78829	-1.727	AA down vs CA	0007166 // cell surface receptor linked signaling pathway // traceable author stat
3404496	KLRF1	NM_016523 // KLR	GGACAATTT	3.38289	chr12:9980076-9997601 (+) //	-0.78829	-1.727	AA down vs CA	0007166 // cell surface receptor linked signaling pathway // traceable author stat
2691982	KPNA1	NR_026698 // KPN	GGAATGTCC	1.62036	chr3:122140657-122233775 (-)	0.828737	1.77613	AA up vs CA	0000018 // regulation of DNA recombination // traceable author statement /// 0
2691982	KPNA1	NR_026698 // KPN	GGAATGTCC	1.62036	chr3:122140657-122233775 (-)	0.828737	1.77613	AA up vs CA	0000018 // regulation of DNA recombination // traceable author statement /// 0
2691982	KPNA1	NR_026698 // KPN	GGAATGTCC	1.62036	chr3:122140657-122233775 (-)	0.828737	1.77613	AA up vs CA	0000018 // regulation of DNA recombination // traceable author statement /// 0
2691982	KPNA1	NR_026698 // KPN	GGAATGTCC	1.62036	chr3:122140657-122233775 (-)	0.828737	1.77613	AA up vs CA	0000018 // regulation of DNA recombination // traceable author statement /// 0
2691982	KPNA1	NR_026698 // KPN	GGAATGTCC	1.62036	chr3:122140657-122233775 (-)	0.828737	1.77613	AA up vs CA	0000018 // regulation of DNA recombination // traceable author statement /// 0
2691982	KPNA1	NR_026698 // KPN	GGAATGTCC	1.62036	chr3:122140657-122233775 (-)	0.828737	1.77613	AA up vs CA	0000018 // regulation of DNA recombination // traceable author statement /// 0
2691982	KPNA1	NR_026698 // KPN	GGAATGTCC	1.62036	chr3:122145616-122233775 (-)	0.828737	1.77613	AA up vs CA	0000018 // regulation of DNA recombination // traceable author statement /// 0
2691982	KPNA1	NR_026698 // KPN	GGAATGTCC	1.62036	chr3:122145616-122233775 (-)	0.828737	1.77613	AA up vs CA	0000018 // regulation of DNA recombination // traceable author statement /// 0
2691982	KPNA1	NR_026698 // KPN	GGAATGTCC	1.62036	chr3:122144526-122233645 (-)	0.828737	1.77613	AA up vs CA	0000018 // regulation of DNA recombination // traceable author statement /// 0
2691982	KPNA1	NR_026698 // KPN	GGAATGTCC	1.62036	chr3:122144526-122233645 (-)	0.828737	1.77613	AA up vs CA	0000018 // regulation of DNA recombination // traceable author statement /// 0
2691982	KPNA1	NR_026698 // KPN	GGAATGTCC	1.62036	chr3:122144525-122182888 (-)	0.828737	1.77613	AA up vs CA	0000018 // regulation of DNA recombination // traceable author statement /// 0

2691982	KPNA1	NR_026698 // KPN/	GGAATGTCC	1.62036	chr3:122144525-122182888 (-)	0.828737	1.77613	AA up vs CA	0000018 // regulation of DNA recombination // traceable author statement /// 0
3756979	KRT38	NM_006771 // KRT	TCAATGTCC	1.72955	chr17:39592620-39597596 (-) /	-0.83041	-1.7782	AA down vs CA	---
2404158	LAPTM5	NM_006762 // LAP	GGTCTGTCC	4.99562	chr1:31205315-31230673 (-) //	0.790305	1.72944	AA up vs CA	0006810 // transport // inferred from electronic annotation
2404158	LAPTM5	NM_006762 // LAP	GCCCTGTCC	4.94793	chr1:31205315-31230673 (-) //	0.790305	1.72944	AA up vs CA	0006810 // transport // inferred from electronic annotation
2404158	LAPTM5	NM_006762 // LAP	GGTCTGTCC	4.99562	chr1:31205315-31230667 (-) //	0.790305	1.72944	AA up vs CA	0006810 // transport // inferred from electronic annotation
2404158	LAPTM5	NM_006762 // LAP	GCCCTGTCC	4.94793	chr1:31205315-31230667 (-) //	0.790305	1.72944	AA up vs CA	0006810 // transport // inferred from electronic annotation
2757427	LETM1	NM_012318 // LETI	GCCCTGTCC	4.94793	chr4:1814759-1857974 (-) // 96	0.806308	1.74873	AA up vs CA	0042407 // cristae formation // inferred from mutant phenotype
2757427	LETM1	NM_012318 // LETI	GCCCTGTCC	4.94793	chr4:1813205-1813866 (-) // 51	0.806308	1.74873	AA up vs CA	0042407 // cristae formation // inferred from mutant phenotype
2949299	LY6G6C	NM_025261 // LY6	GGACACTGT	2.37683	chr6:31686424-31689510 (-) //	-0.78974	-1.7288	AA down vs CA	---
2949299	LY6G6C	NM_025261 // LY6	GGACAATTG	3.81475	chr6:31686424-31689510 (-) //	-0.78974	-1.7288	AA down vs CA	---
3753935	LYZL6	NM_020426 // LYZI	GGACATTTA	2.73561	chr17:34261524-34266510 (-) /	-0.92692	-1.9012	AA down vs CA	0008152 // metabolic process // inferred from electronic annotation /// 0016998
3994710	MAMLD1	NM_005491 // MAN	GAATTGTCC	1.65542	chrX:149613719-149682439 (+)	-0.80224	-1.7438	AA down vs CA	0006350 // transcription // inferred from electronic annotation /// 0008584 // r
2376922	MAPKAPI	NM_004759 // MAF	TCACTGTCC	0.86373	chr1:206858288-206907628 (+)	0.832898	1.78126	AA up vs CA	0000165 // MAPKKK cascade // traceable author statement /// 0006468 // prot
2376922	MAPKAPI	NM_004759 // MAF	TCACTGTCC	0.86373	chr1:206858288-206906208 (+)	0.832898	1.78126	AA up vs CA	0000165 // MAPKKK cascade // traceable author statement /// 0006468 // prot
2376922	MAPKAPI	NM_004759 // MAF	TCACTGTCC	0.86373	chr1:206858586-206906610 (+)	0.832898	1.78126	AA up vs CA	0000165 // MAPKKK cascade // traceable author statement /// 0006468 // prot
2922215	MARCKS	NM_002356 // MAF	GGACATTAA	2.73561	chr6:114178526-114182981 (+)	0.893634	1.85785	AA up vs CA	---
2922215	MARCKS	NM_002356 // MAF	GGACATTAA	2.73561	chr6:114178555-114182980 (+)	0.893634	1.85785	AA up vs CA	---
2922215	MARCKS	NM_002356 // MAF	GGACATTAA	2.73561	chr6:114178616-114182318 (+)	0.893634	1.85785	AA up vs CA	---
2922215	MARCKS	NM_002356 // MAF	GGACATTAA	2.73561	chr6:114181655-114182980 (-)	0.893634	1.85785	AA up vs CA	---
2934274	MAS1	NM_002377 // MAS	CCATTGTCC	2.8087	chr6:160327973-160329108 (+)	-0.79592	-1.7362	AA down vs CA	0001933 // negative regulation of protein phosphorylation // inferred from electro
2831124	MATR3	NM_199189 // MAT	GGACAATTA	2.51915	chr5:138629379-138666533 (+)	0.836571	1.7858	AA up vs CA	---
2831124	MATR3	NM_199189 // MAT	GGACAATTA	2.51915	chr5:138642851-138666098 (+)	0.836571	1.7858	AA up vs CA	---
2831124	MATR3	NM_199189 // MAT	GGACAATTA	2.51915	chr5:138665805-138666126 (+)	0.836571	1.7858	AA up vs CA	---
3702293	MBTPS1	NM_003791 // MBT	GGACACTTC	1.65542	chr16:84087370-84150511 (-) /	0.802127	1.74367	AA up vs CA	0006508 // proteolysis // inferred from electronic annotation /// 0006508 // pro
3702293	MBTPS1	NM_003791 // MBT	GGACACTTC	1.65542	chr16:84092973-84095209 (-) /	0.802127	1.74367	AA up vs CA	0006508 // proteolysis // inferred from electronic annotation /// 0006508 // pro
3063685	MCM7	NM_005916 // MCN	GCGGTGTCC	4.89044	chr7:99690403-99699033 (-) //	0.807907	1.75067	AA up vs CA	0006260 // DNA replication // not recorded /// 0006260 // DNA replication // ir
3063685	MCM7	NM_005916 // MCN	GCGGTGTCC	4.89044	chr7:99690918-99697360 (-) //	0.807907	1.75067	AA up vs CA	0006260 // DNA replication // not recorded /// 0006260 // DNA replication // ir
2420681	MCOLN3	NM_018298 // MCC	CGACTGTCC	2.91388	chr1:85484590-85514129 (-) //	-0.87652	-1.8359	AA down vs CA	0006810 // transport // inferred from electronic annotation /// 0006811 // ion t
3461164	MDM1	NM_017440 // MDN	GGACAATTT	3.38289	chr12:68688345-68715402 (-) /	-1.13471	-2.1957	AA down vs CA	---
3461164	MDM1	NM_017440 // MDN	GGACAATTT	3.38289	chr12:68718900-68726083 (-) /	-1.13471	-2.1957	AA down vs CA	---
3939545	MIF	NM_002415 // MIF	GGACACTCA	2.26764	chr22:24236564-24237409 (+) ,	0.861773	1.81727	AA up vs CA	0001516 // prostaglandin biosynthetic process // inferred from direct assay /// 0
2950885	MLN	NM_002418 // MLN	ACATTGTCC	2.37683	chr6:33762448-33771793 (-) //	-0.99168	-1.9885	AA down vs CA	0007186 // G-protein coupled receptor protein signaling pathway // traceable aut
2488078	MPHOSPI	NM_005791 // MPH	GGACATTAG	4.03121	chr2:71357500-71377230 (+) //	0.841699	1.79216	AA up vs CA	0000375 // RNA splicing, via transesterification reactions // non-traceable author
2488078	MPHOSPI	NM_005791 // MPH	CGAATGTCC	3.77969	chr2:71357500-71377230 (+) //	0.841699	1.79216	AA up vs CA	0000375 // RNA splicing, via transesterification reactions // non-traceable author
2674919	MST1R	NM_002447 // MST	GGACAGTCC	0.75454	chr3:49924437-49941070 (-) //	-0.82821	-1.7755	AA down vs CA	0006468 // protein phosphorylation // inferred from electronic annotation /// 00
2674919	MST1R	NM_002447 // MST	CCAGTGTCC	2.8087	chr3:49924437-49941070 (-) //	-0.82821	-1.7755	AA down vs CA	0006468 // protein phosphorylation // inferred from electronic annotation /// 00

2980258	MTRF1L	NM_019041 // MTR	GCTGTGTCC	4.89044	chr6:153322627-153323824 (-)	-0.94676	-1.9275	AA down vs CA	0006412 // translation // inferred from electronic annotation /// 0006415 // tra
2980258	MTRF1L	NM_019041 // MTR	GCTGTGTCC	4.89044	chr6:153319433-153320643 (-)	-0.94676	-1.9275	AA down vs CA	0006412 // translation // inferred from electronic annotation /// 0006415 // tra
2451567	MYBPH	NM_004997 // MYB	CGATTGTCC	3.56324	chr1:203136938-203144911 (-)	-0.91109	-1.8805	AA down vs CA	0006942 // regulation of striated muscle contraction // traceable author stateme
2931970	MYCT1	NM_025107 // MYC	GGACATTGT	2.59328	chr6:153019037-153043758 (+)	-0.97504	-1.9657	AA down vs CA	---
3678516	NAGPA	NM_016256 // NAG	GGACACTGC	0.64936	chr16:5074846-5083915 (-) // 9	0.780167	1.71733	AA up vs CA	0005975 // carbohydrate metabolic process // traceable author statement /// 00
3678516	NAGPA	NM_016256 // NAG	TAAGTGTCC	2.51915	chr16:5074846-5083915 (-) // 9	0.780167	1.71733	AA up vs CA	0005975 // carbohydrate metabolic process // traceable author statement /// 00
3678516	NAGPA	NM_016256 // NAG	GGACAATTG	3.81475	chr16:5074846-5083915 (-) // 9	0.780167	1.71733	AA up vs CA	0005975 // carbohydrate metabolic process // traceable author statement /// 00
3326252	NAT10	NM_024662 // NAT	GGACACTAT	3.38289	chr11:34127148-34168457 (+) /	0.820134	1.76557	AA up vs CA	0008152 // metabolic process // inferred from electronic annotation
2603460	NCL	NM_005381 // NCL	GGACACTGG	2.8087	chr2:232319618-232329175 (-)	0.843542	1.79445	AA up vs CA	0001525 // angiogenesis // inferred from direct assay
2322389	NECAP2	NM_018090 // NEC	GGTCTGTCC	4.99562	chr1:16767237-16786572 (+) //	0.892461	1.85634	AA up vs CA	0006810 // transport // inferred from electronic annotation /// 0006897 // endo
3044518	NEUROD6	NM_022728 // NEU	GGACACTGA	1.5131	chr7:31377694-31380340 (-) //	-0.79259	-1.7322	AA down vs CA	0006350 // transcription // inferred from electronic annotation /// 0007275 // r
3044518	NEUROD6	NM_022728 // NEU	GGACAATAA	2.51915	chr7:31377694-31380340 (-) //	-0.79259	-1.7322	AA down vs CA	0006350 // transcription // inferred from electronic annotation /// 0007275 // r
3572975	NGB	NM_021257 // NGB	GCCCTGTCC	4.94793	chr14:77732816-77737280 (-) /	-0.84928	-1.8016	AA down vs CA	0006810 // transport // inferred from electronic annotation /// 0015671 // oxyg
2603544	NMUR1	NM_006056 // NMU	GGACAGTAT	2.73353	chr2:232389753-232393662 (-)	-0.88623	-1.8483	AA down vs CA	0006816 // calcium ion transport // inferred from direct assay /// 0006821 // cl
2603544	NMUR1	NM_006056 // NMU	GGACAATGG	2.8087	chr2:232389753-232393662 (-)	-0.88623	-1.8483	AA down vs CA	0006816 // calcium ion transport // inferred from direct assay /// 0006821 // cl
3980887	NONO	NM_001145408 //	GGACACTCC	1.4039	chrX:70503501-70521016 (+) //	0.816051	1.76058	AA up vs CA	0006281 // DNA repair // inferred from electronic annotation /// 0006310 // DN
3980887	NONO	NM_001145408 //	GGACACTCC	1.4039	chrX:70503501-70519992 (+) //	0.816051	1.76058	AA up vs CA	0006281 // DNA repair // inferred from electronic annotation /// 0006310 // DN
3980887	NONO	NM_001145408 //	GGACACTCC	1.4039	chrX:70503504-70520118 (+) //	0.816051	1.76058	AA up vs CA	0006281 // DNA repair // inferred from electronic annotation /// 0006310 // DN
2402883	NR0B2	NM_021969 // NR0	GGACAGTGG	2.15934	chr1:27237978-27240460 (-) //	-0.92206	-1.8948	AA down vs CA	0000122 // negative regulation of transcription from RNA polymerase II promoter
2540157	ODC1	NM_002539 // ODC	GGATTGTCC	1.4039	chr2:10580507-10588453 (-) //	0.820632	1.76618	AA up vs CA	0001822 // kidney development // inferred from electronic annotation /// 00065
2540157	ODC1	NM_002539 // ODC	AAAGTGTCC	3.38289	chr2:10580507-10588453 (-) //	0.820632	1.76618	AA up vs CA	0001822 // kidney development // inferred from electronic annotation /// 00065
2947789	OR12D3	NM_030959 // OR1	GGACATTGT	2.59328	chr6:29342113-29343064 (-) //	-0.83145	-1.7795	AA down vs CA	0007165 // signal transduction // inferred from electronic annotation /// 00718
2947789	OR12D3	NM_030959 // OR1	GGACAGCTT	6.97461	chr6:29342113-29343064 (-) //	-0.83145	-1.7795	AA down vs CA	0007165 // signal transduction // inferred from electronic annotation /// 00718
2947789	OR12D3	NM_030959 // OR1	GGACAGTGG	2.15934	chr6:29342113-29343064 (-) //	-0.83145	-1.7795	AA down vs CA	0007165 // signal transduction // inferred from electronic annotation /// 00718
2900143	OR2B6	NM_012367 // OR2	GGACAGTAT	2.73353	chr6:27925018-27925960 (+) //	-0.8691	-1.8265	AA down vs CA	0007165 // signal transduction // inferred from electronic annotation /// 00718
2407934	PABPC4	NM_001135653 //	TAAATGTCC	2.73561	chr1:40026487-40042416 (-) //	0.954315	1.93766	AA up vs CA	0006396 // RNA processing // traceable author statement /// 0006401 // RNA c
2728189	PAICS	NM_001079525 //	GCGCTGTCC	4.24108	chr4:57302322-57327527 (+) //	0.855934	1.80993	AA up vs CA	0006164 // purine nucleotide biosynthetic process // inferred from electronic ann
2728189	PAICS	NM_001079525 //	GCGCTGTCC	4.24108	chr4:57302454-57325828 (+) //	0.855934	1.80993	AA up vs CA	0006164 // purine nucleotide biosynthetic process // inferred from electronic ann
2728189	PAICS	NM_001079525 //	GCGCTGTCC	4.24108	chr4:57319873-57327970 (+) //	0.855934	1.80993	AA up vs CA	0006164 // purine nucleotide biosynthetic process // inferred from electronic ann
2780999	PAPSS1	NM_005443 // PAP	AAAATGTCC	3.59934	chr4:108535055-108641361 (-)	1.005228	2.00726	AA up vs CA	0000103 // sulfate assimilation // inferred from electronic annotation /// 00150
2560195	PCGF1	NM_032673 // PCG	AAATTGTCC	3.38289	chr2:74732170-74734786 (-) //	0.791281	1.73061	AA up vs CA	0006350 // transcription // inferred from electronic annotation /// 0045449 // r
2560195	PCGF1	NM_032673 // PCG	AAATTGTCC	3.38289	chr2:74732170-74734786 (-) //	0.791281	1.73061	AA up vs CA	0006350 // transcription // inferred from electronic annotation /// 0045449 // r
2633930	PCNP	NM_020357 // PCN	GGACAATCG	3.56324	chr3:101293041-101313279 (+)	0.844707	1.7959	AA up vs CA	0007049 // cell cycle // inferred from electronic annotation /// 0016567 // prot
3393200	PCSK7	NM_004716 // PCS	GGACAATGG	2.8087	chr11:117075811-117101185 (-	1.002724	2.00378	AA up vs CA	0006508 // proteolysis // inferred from electronic annotation /// 0016486 // pe
3044597	PDE1C	NM_001191059 //	GGACAGTGG	2.15934	chr7:31829246-32110991 (-) //	-0.80355	-1.7454	AA down vs CA	0006198 // cAMP catabolic process // inferred from electronic annotation /// 00
3044597	PDE1C	NM_001191059 //	GGACAGTGG	2.15934	chr7:31792617-31917640 (-) //	-0.80355	-1.7454	AA down vs CA	0006198 // cAMP catabolic process // inferred from electronic annotation /// 00

3381150	PDE2A	NR_026572 // PDE2	TTACTGTCC	1.86979	chr11:72287185-72385412 (-) /	-0.97962	-1.972	AA down vs CA	0006198 // cAMP catabolic process // inferred from electronic annotation /// 00(
3406229	PDE6H	NM_006205 // PDE	TAAGTGTCC	2.51915	chr12:15125977-15134799 (+) /	-0.8175	-1.7624	AA down vs CA	0000187 // activation of MAPK activity // inferred from electronic annotation ///
3406229	PDE6H	NM_006205 // PDE	TCAATGTCC	1.72955	chr12:15125977-15134799 (+) /	-0.8175	-1.7624	AA down vs CA	0000187 // activation of MAPK activity // inferred from electronic annotation ///
2598606	PECR	NM_018441 // PEC	AAAGTGTCC	3.38289	chr2:216903826-216946521 (-)	-0.94424	-1.9242	AA down vs CA	0006631 // fatty acid metabolic process // traceable author statement /// 0006
3744150	PER1	NM_002616 // PER	AGAATGTCC	3.34783	chr17:8043789-8055702 (-) // 9	-1.11459	-2.1653	AA down vs CA	0006350 // transcription // inferred from electronic annotation /// 0006355 // r
3744150	PER1	NM_002616 // PER	AGAATGTCC	3.34783	chr17:8043790-8055702 (-) // 8	-1.11459	-2.1653	AA down vs CA	0006350 // transcription // inferred from electronic annotation /// 0006355 // r
2318656	PER3	NM_016831 // PER	GGACACTCC	1.4039	chr1:7844762-7905237 (+) // 9	-0.98538	-1.9798	AA down vs CA	0006350 // transcription // inferred from electronic annotation /// 0006355 // r
3403299	PEX5	NM_001131025 //	GGTCTGTCC	4.99562	chr12:7342959-7364044 (+) //	-0.94185	-1.921	AA down vs CA	0000038 // very long-chain fatty acid metabolic process // inferred from electron
3403299	PEX5	NM_001131025 //	GGTCTGTCC	4.99562	chr12:7342956-7371170 (+) //	-0.94185	-1.921	AA down vs CA	0000038 // very long-chain fatty acid metabolic process // inferred from electron
3742400	PFN1	NM_005022 // PFN	GGACAATCC	1.4039	chr1:171639419-171640218 (-)	0.830012	1.7777	AA up vs CA	0001843 // neural tube closure // inferred from electronic annotation /// 00063!
2460189	PGBD5	NM_024554 // PGB	GGACAGTGA	0.86373	chr1:230457404-230468617 (-)	-0.93715	-1.9147	AA down vs CA	---
3867865	PIH1D1	NM_017916 // PIH1	GGACATTGA	1.72955	chr19:49949555-49955067 (-) /	0.79526	1.73539	AA up vs CA	0000492 // box C/D snoRNP assembly // inferred from mutant phenotype
2697564	PIK3CB	NM_006219 // PIK3	TAAGTGTCC	2.51915	chr3:138372861-138515409 (-)	0.847003	1.79876	AA up vs CA	0000187 // activation of MAPK activity // traceable author statement /// 00019
2697564	PIK3CB	NM_006219 // PIK3	TAAGTGTCC	2.51915	chr3:138471722-138474823 (-)	0.847003	1.79876	AA up vs CA	0000187 // activation of MAPK activity // traceable author statement /// 00019
2876361	PITX1	NM_002653 // PITX	GGACAAAGC	4.89044	chr5:134364467-134370085 (-)	-0.82905	-1.7765	AA down vs CA	0001501 // skeletal system development // traceable author statement /// 0001
2876361	PITX1	NM_002653 // PITX	GGACAAAGC	4.89044	chr5:134363468-134369774 (-)	-0.82905	-1.7765	AA down vs CA	0001501 // skeletal system development // traceable author statement /// 0001
2638077	PLA1A	NM_015900 // PLA	GGACACTTA	2.51915	chr3:119316744-119348651 (+)	0.971891	1.96141	AA up vs CA	0006629 // lipid metabolic process // traceable author statement /// 0006629 /
2638077	PLA1A	NM_015900 // PLA	GGACAGTGT	1.72747	chr3:119316744-119348651 (+)	0.971891	1.96141	AA up vs CA	0006629 // lipid metabolic process // traceable author statement /// 0006629 /
3142362	PMP2	NM_002677 // PMP	CTAATGTCC	4.03121	chr8:82354026-82359655 (-) //	-0.81549	-1.7599	AA down vs CA	0006810 // transport // inferred from electronic annotation
3818897	PNPLA6	NM_001166111 //	GGACAGCCC	4.99562	chr19:7599750-7626647 (+) //	0.788903	1.72776	AA up vs CA	0001525 // angiogenesis // inferred from electronic annotation /// 0006629 // l
2770242	PPAT	NM_002703 // PPA	GGACAGCGC	4.24108	chr3:87098347-87102016 (-) //	0.907721	1.87608	AA up vs CA	0006164 // purine nucleotide biosynthetic process // inferred from electronic ann
2770242	PPAT	NM_002703 // PPA	GGACAGCGC	4.24108	chr3:87098355-87101938 (-) //	0.907721	1.87608	AA up vs CA	0006164 // purine nucleotide biosynthetic process // inferred from electronic ann
3090922	PPP2R2A	NM_002717 // PPP:	TTAGTGTCC	2.51915	chr8:26149230-26228610 (+) //	0.788051	1.72674	AA up vs CA	0006470 // protein dephosphorylation // inferred from direct assay /// 0007165
3090922	PPP2R2A	NM_002717 // PPP:	GGACACTGC	0.64936	chr8:26149230-26228610 (+) //	0.788051	1.72674	AA up vs CA	0006470 // protein dephosphorylation // inferred from direct assay /// 0007165
3373811	PRG2	NM_002728 // PRG	TCAGTGTCC	1.5131	chr11:57154842-57158123 (-) /	-0.79494	-1.735	AA down vs CA	0006955 // immune response // inferred from electronic annotation /// 0042742
2379665	PROX1	NM_002763 // PRO	GGACAATAT	3.38289	chr1:214161285-214209282 (+)	-0.91763	-1.889	AA down vs CA	0000122 // negative regulation of transcription from RNA polymerase II promoter
3893849	PRPF6	NM_012469 // PRPI	AGAATGTCC	3.34783	chr20:62612448-62664453 (+) /	0.950752	1.93288	AA up vs CA	0000244 // assembly of spliceosomal tri-snRNP // inferred by curator /// 000024
3893849	PRPF6	NM_012469 // PRPI	GGACAGGGC	4.94793	chr20:62612448-62664453 (+) /	0.950752	1.93288	AA up vs CA	0000244 // assembly of spliceosomal tri-snRNP // inferred by curator /// 000024
3893849	PRPF6	NM_012469 // PRPI	AGAATGTCC	3.34783	chr20:62612516-62664416 (+) /	0.950752	1.93288	AA up vs CA	0000244 // assembly of spliceosomal tri-snRNP // inferred by curator /// 000024
3893849	PRPF6	NM_012469 // PRPI	GGACAGGGC	4.94793	chr20:62612516-62664416 (+) /	0.950752	1.93288	AA up vs CA	0000244 // assembly of spliceosomal tri-snRNP // inferred by curator /// 000024
2903285	PSMB9	NM_002800 // PSM	GTAGTGTCC	1.65542	chr6:32821961-32827327 (+) //	0.791414	1.73077	AA up vs CA	0005975 // carbohydrate metabolic process // inferred from electronic annotatior
2903285	PSMB9	NM_002800 // PSM	GGACAGTTA	1.86979	chr6:32821961-32827327 (+) //	0.791414	1.73077	AA up vs CA	0005975 // carbohydrate metabolic process // inferred from electronic annotatior
3557811	PSME2	NM_002818 // PSM	GCTCTGTCC	4.24108	chr13:49345231-49346006 (+) /	0.798133	1.73885	AA up vs CA	0031145 // anaphase-promoting complex-dependent proteasomal ubiquitin-depen
2948810	PSORS1C	NM_014069 // PSO	ACACTGTCC	1.72747	chr6:31105310-31107118 (-) //	-0.81319	-1.7571	AA down vs CA	---
2556667	RAB1A	NM_004161 // RAB	GCTGTGTCC	4.89044	chr2:65315570-65357083 (-) //	0.785927	1.7242	AA up vs CA	0006810 // transport // inferred from electronic annotation /// 0007264 // sma
2556667	RAB1A	NM_004161 // RAB	GCTGTGTCC	4.89044	chr2:65315013-65357227 (-) //	0.785927	1.7242	AA up vs CA	0006810 // transport // inferred from electronic annotation /// 0007264 // sma

2556667	RAB1A	NM_004161 // RAB	GCTGTGTCC	4.89044	chr2:65313553-65315061 (-) //	0.785927	1.7242	AA up vs CA	0006810 // transport // inferred from electronic annotation /// 0007264 // sma
2534509	RAMP1	NM_005855 // RAM	CGAGTGTCC	3.56324	chr2:238768286-238820746 (+)	0.858252	1.81284	AA up vs CA	0006810 // transport // inferred from electronic annotation /// 0006886 // intra
2534509	RAMP1	NM_005855 // RAM	GCCCTGTCC	4.94793	chr2:238768286-238820746 (+)	0.858252	1.81284	AA up vs CA	0006810 // transport // inferred from electronic annotation /// 0006886 // intra
3333899	RARRES3	NM_004585 // RAR	ACAATGTCC	2.59328	chr11:63304279-63313929 (+) /	0.834986	1.78384	AA up vs CA	0008285 // negative regulation of cell proliferation // traceable author statement
3744958	RCVRN	NM_002903 // RCV	GGACAATAA	2.51915	chr17:9801027-9808684 (-) // 9	-0.82855	-1.7759	AA down vs CA	0007165 // signal transduction // traceable author statement /// 0007601 // vis
3744958	RCVRN	NM_002903 // RCV	AGAGTGTCC	3.13137	chr17:9801027-9808684 (-) // 9	-0.82855	-1.7759	AA down vs CA	0007165 // signal transduction // traceable author statement /// 0007601 // vis
3852407	RFX1	NM_002918 // RFX	GGACATTCA	2.48409	chr19:14073453-14116954 (-) /	-0.7983	-1.7391	AA down vs CA	0006350 // transcription // inferred from electronic annotation /// 0006355 // r
2674242	RHOA	NM_001664 // RHC	GGACTGTCC	0.75454	chr3:49396929-49449362 (-) //	0.920697	1.89303	AA up vs CA	0000902 // cell morphogenesis // inferred from electronic annotation /// 00016
2674242	RHOA	NM_001664 // RHC	GGAGTGTCC	1.4039	chr3:49396929-49449362 (-) //	0.920697	1.89303	AA up vs CA	0000902 // cell morphogenesis // inferred from electronic annotation /// 00016
2384401	RHOU	NM_021205 // RHC	AAAGTGTCC	3.38289	chr4:13607404-13662838 (-)	0.784253	1.7222	AA up vs CA	---
2384401	RHOU	NM_021205 // RHC	GGACACTTC	1.65542	chr4:13607404-13662838 (-)	0.784253	1.7222	AA up vs CA	---
3527722	RNASE2	NM_002934 // RNA	GCTCTGTCC	4.24108	chr14:21423629-21424594 (+) /	-1.1455	-2.2122	AA down vs CA	0006401 // RNA catabolic process // traceable author statement /// 0006935 //
3527722	RNASE2	NM_002934 // RNA	CAACTGTCC	3.16539	chr14:21423629-21424594 (+) /	-1.1455	-2.2122	AA down vs CA	0006401 // RNA catabolic process // traceable author statement /// 0006935 //
3527722	RNASE2	NM_002934 // RNA	GGACAGTTA	1.86979	chr14:21423629-21424594 (+) /	-1.1455	-2.2122	AA down vs CA	0006401 // RNA catabolic process // traceable author statement /// 0006935 //
3527722	RNASE2	NM_002934 // RNA	GGGCTGTCC	4.99562	chr14:21423629-21424594 (+) /	-1.1455	-2.2122	AA down vs CA	0006401 // RNA catabolic process // traceable author statement /// 0006935 //
3396198	ROBO4	NM_019055 // ROB	GCTGTGTCC	4.89044	chr11:124756282-124763841 (-	-0.82157	-1.7673	AA down vs CA	0001525 // angiogenesis // inferred from genetic interaction /// 0001525 // ang
2417500	RPE65	NM_000329 // RPE	GGACAATAC	1.65542	chr1:68894444-68915642 (-) //	-1.00157	-2.0022	AA down vs CA	0006776 // vitamin A metabolic process // traceable author statement /// 0007
2614120	RPL15	NM_002948 // RPL	GGACACTCC	1.4039	chr3:23959350-23960992 (+) //	0.81628	1.76086	AA up vs CA	0006412 // translation // traceable author statement /// 0006412 // translation
2614120	RPL15	NM_002948 // RPL	GGACACTCC	1.4039	chr3:23958614-23962467 (+) //	0.81628	1.76086	AA up vs CA	0006412 // translation // traceable author statement /// 0006412 // translation
2614120	RPL15	NM_002948 // RPL	GGACACTCC	1.4039	chr3:23959337-23962330 (+) //	0.81628	1.76086	AA up vs CA	0006412 // translation // traceable author statement /// 0006412 // translation
2694397	RPN1	NM_002950 // RPN	GGACAGGGC	4.94793	chr3:128338818-128370048 (-)	0.793563	1.73335	AA up vs CA	0006464 // protein modification process // traceable author statement /// 0006
2844082	RUFY1	NM_025158 // RUF	CAACTGTCC	3.16539	chr5:179012794-179037012 (+)	0.815485	1.75989	AA up vs CA	0006810 // transport // inferred from electronic annotation /// 0006897 // endo
3844978	SBNO2	NM_014963 // SBN	ATATTGTCC	3.38289	chr19:1107657-1174259 (-) // 9	0.860724	1.81595	AA up vs CA	0002281 // macrophage activation involved in immune response // inferred from r
3844978	SBNO2	NM_014963 // SBN	ATATTGTCC	3.38289	chr19:1108218-1132133 (-) // 9	0.860724	1.81595	AA up vs CA	0002281 // macrophage activation involved in immune response // inferred from r
2662657	SEC13	NR_024272 // SEC	GGACAGTCT	2.48201	chr3:10342616-10360857 (-) //	0.928556	1.90337	AA up vs CA	0006810 // transport // inferred from electronic annotation /// 0006886 // intra
2783316	SEC24D	NM_014822 // SEC	CCACTGTCC	2.15934	chr4:119643978-119757254 (-)	0.815952	1.76046	AA up vs CA	0006810 // transport // inferred from electronic annotation /// 0006886 // intra
2783316	SEC24D	NM_014822 // SEC	CCACTGTCC	2.15934	chr4:119657199-119659093 (-)	0.815952	1.76046	AA up vs CA	0006810 // transport // inferred from electronic annotation /// 0006886 // intra
2783316	SEC24D	NM_014822 // SEC	CCACTGTCC	2.15934	chr4:119650285-119654470 (-)	0.815952	1.76046	AA up vs CA	0006810 // transport // inferred from electronic annotation /// 0006886 // intra
3946380	SGSM3	NM_015705 // SGS	GGACACTTA	2.51915	chr22:40796699-40806115 (+) /	0.810147	1.75339	AA up vs CA	0007049 // cell cycle // inferred from electronic annotation /// 0007050 // cell c
3946380	SGSM3	NM_015705 // SGS	ATACTGTCC	2.73353	chr22:40796699-40806115 (+) /	0.810147	1.75339	AA up vs CA	0007049 // cell cycle // inferred from electronic annotation /// 0007050 // cell c
3946380	SGSM3	NM_015705 // SGS	GGACACTTA	2.51915	chr22:40799917-40806289 (+) /	0.810147	1.75339	AA up vs CA	0007049 // cell cycle // inferred from electronic annotation /// 0007050 // cell c
3946380	SGSM3	NM_015705 // SGS	ATACTGTCC	2.73353	chr22:40799917-40806289 (+) /	0.810147	1.75339	AA up vs CA	0007049 // cell cycle // inferred from electronic annotation /// 0007050 // cell c
3946380	SGSM3	NM_015705 // SGS	GGACACTTA	2.51915	chr22:40802563-40806108 (+) /	0.810147	1.75339	AA up vs CA	0007049 // cell cycle // inferred from electronic annotation /// 0007050 // cell c
3946380	SGSM3	NM_015705 // SGS	ATACTGTCC	2.73353	chr22:40802563-40806108 (+) /	0.810147	1.75339	AA up vs CA	0007049 // cell cycle // inferred from electronic annotation /// 0007050 // cell c
3846667	SIRT6	NM_016539 // SIRT	GCTCTGTCC	4.24108	chr19:4174105-4182596 (-) // 9	0.782459	1.72006	AA up vs CA	0006342 // chromatin silencing // inferred from electronic annotation /// 00064
3266279	SLC18A2	NM_003054 // SLC	GGACACTGA	1.5131	chr10:119000633-119039243 (-	-0.88147	-1.8423	AA down vs CA	0001975 // response to amphetamine // inferred from electronic annotation /// 0

3266279	SLC18A2	NM_003054 // SLC	AAAGTGTCC	3.38289	chr10:119000633-119039243 (-	-0.88147	-1.8423	AA down vs CA	0001975 // response to amphetamine // inferred from electronic annotation /// 0
3452417	SLC38A4	NM_018018 // SLC	GGACAGTAT	2.73353	chr12:47158543-47219768 (-) /	-0.94109	-1.92	AA down vs CA	0006810 // transport // inferred from electronic annotation /// 0006811 // ion t
3888383	SLC9A8	NM_015266 // SLC	GGACAATAG	3.81475	chr20:48466115-48508774 (+) /	-0.99729	-1.9962	AA down vs CA	0006810 // transport // inferred from electronic annotation /// 0006811 // ion t
3888383	SLC9A8	NM_015266 // SLC	GAAGTGTCC	1.65542	chr20:48466115-48508774 (+) /	-0.99729	-1.9962	AA down vs CA	0006810 // transport // inferred from electronic annotation /// 0006811 // ion t
3779612	SLMO1	NM_001142405 //	GGACAGGGC	4.94793	chr18:12431580-12432232 (+) /	-0.9553	-1.939	AA down vs CA	---
3779612	SLMO1	NM_001142405 //	GGACAGTGG	2.15934	chr18:12431580-12432232 (+) /	-0.9553	-1.939	AA down vs CA	---
3456592	SMUG1	NM_014311 // SML	CCACTGTCC	2.15934	chr12:54575251-54581692 (-) /	0.915498	1.88622	AA up vs CA	0006281 // DNA repair // inferred from electronic annotation /// 0006284 // bas
3456592	SMUG1	NM_014311 // SML	GGACAGTAA	1.86979	chr12:54575251-54581692 (-) /	0.915498	1.88622	AA up vs CA	0006281 // DNA repair // inferred from electronic annotation /// 0006284 // bas
3238528	SPAG6	NM_012443 // SPA	GGACATTCA	2.48409	chr10:22634415-22706539 (+) /	-0.93631	-1.9136	AA down vs CA	0007286 // spermatid development // traceable author statement /// 0030030 /
3238528	SPAG6	NM_012443 // SPA	GGACATTCA	2.48409	chr10:22634415-22706536 (+) /	-0.93631	-1.9136	AA down vs CA	0007286 // spermatid development // traceable author statement /// 0030030 /
2528504	SPEG	NM_005876 // SPE	GGAATGTCC	1.62036	chr2:220326602-220331583 (+)	-0.793	-1.7327	AA down vs CA	0006468 // protein phosphorylation // inferred from electronic annotation /// 00
2756965	SPON2	NM_012445 // SPO	GCTCTGTCC	4.24108	chr4:1160722-1166597 (-) // 98	0.881273	1.842	AA up vs CA	0007155 // cell adhesion // inferred from electronic annotation /// 0007411 // a
3568485	SPTB	NM_001024858 //	GGAATGTCC	1.62036	chr14:65233117-65289866 (-) /	-0.81633	-1.7609	AA down vs CA	0006779 // porphyrin biosynthetic process // inferred from electronic annotation
3568485	SPTB	NM_001024858 //	GGAATGTCC	1.62036	chr14:65212907-65213887 (-) /	-0.81633	-1.7609	AA down vs CA	0006779 // porphyrin biosynthetic process // inferred from electronic annotation
2662087	SRGAP3	NM_014850 // SRG	ATAATGTCC	3.59934	chr3:9022277-9094759 (-) // 94	-0.80225	-1.7438	AA down vs CA	0007165 // signal transduction // inferred from electronic annotation
2662087	SRGAP3	NM_014850 // SRG	ATAATGTCC	3.59934	chr3:9048643-9052939 (-) // 62	-0.80225	-1.7438	AA down vs CA	0007165 // signal transduction // inferred from electronic annotation
2396461	SRM	NM_003132 // SRM	GGACACTGC	0.64936	chr1:11114676-11120081 (-) //	0.895349	1.86006	AA up vs CA	0008295 // spermidine biosynthetic process // inferred from electronic annotation
3396916	SRPR	NM_003139 // SRP	CCACTGTCC	2.15934	chr11:126132829-126138809 (-	0.793372	1.73312	AA up vs CA	0006613 // cotranslational protein targeting to membrane // traceable author sta
3396916	SRPR	NM_003139 // SRP	CCACTGTCC	2.15934	chr11:126132829-126138708 (-	0.793372	1.73312	AA up vs CA	0006613 // cotranslational protein targeting to membrane // traceable author sta
3643552	SSTR5	NM_001053 // SST	AAAATGTCC	3.59934	chr16:1128837-1130085 (+) //	-0.81391	-1.758	AA down vs CA	0007165 // signal transduction // inferred from electronic annotation /// 00718
3643552	SSTR5	NM_001053 // SST	GGAGTGTCC	1.4039	chr16:1128837-1130085 (+) //	-0.81391	-1.758	AA down vs CA	0007165 // signal transduction // inferred from electronic annotation /// 00718
2633256	ST3GAL6	NM_006100 // ST3	GGACATTAA	2.73561	chr3:98451129-98512805 (+) /	-0.95358	-1.9367	AA down vs CA	0006040 // amino sugar metabolic process // traceable author statement /// 00(
2633256	ST3GAL6	NM_006100 // ST3	GGACATTAA	2.73561	chr3:98451159-98514689 (+) /	-0.95358	-1.9367	AA down vs CA	0006040 // amino sugar metabolic process // traceable author statement /// 00(
2633256	ST3GAL6	NM_006100 // ST3	GGACATTAA	2.73561	chr3:98451159-98514689 (+) /	-0.95358	-1.9367	AA down vs CA	0006040 // amino sugar metabolic process // traceable author statement /// 00(
2592268	STAT1	NM_007315 // STA	TCATTGTCC	1.5131	chr2:191833875-191878405 (-)	1.093756	2.13429	AA up vs CA	0006350 // transcription // inferred from electronic annotation /// 0006355 // r
2592268	STAT1	NM_007315 // STA	TCATTGTCC	1.5131	chr2:191840367-191878894 (-)	1.093756	2.13429	AA up vs CA	0006350 // transcription // inferred from electronic annotation /// 0006355 // r
2592268	STAT1	NM_007315 // STA	TCATTGTCC	1.5131	chr2:191833875-191878405 (-)	1.093756	2.13429	AA up vs CA	0006350 // transcription // inferred from electronic annotation /// 0006355 // r
2680819	SUCLG2	NM_003848 // SUC	CCACTGTCC	2.15934	chr12:94942002-94944344 (+) /	0.84396	1.79497	AA up vs CA	0006099 // tricarboxylic acid cycle // inferred from electronic annotation /// 000
2680819	SUCLG2	NM_003848 // SUC	GGACAGTAG	3.16539	chr12:94942002-94944344 (+) /	0.84396	1.79497	AA up vs CA	0006099 // tricarboxylic acid cycle // inferred from electronic annotation /// 000
2680819	SUCLG2	NM_003848 // SUC	CCACTGTCC	2.15934	chr12:94942031-94944325 (+) /	0.84396	1.79497	AA up vs CA	0006099 // tricarboxylic acid cycle // inferred from electronic annotation /// 000
2680819	SUCLG2	NM_003848 // SUC	GGACAGTAG	3.16539	chr12:94942031-94944325 (+) /	0.84396	1.79497	AA up vs CA	0006099 // tricarboxylic acid cycle // inferred from electronic annotation /// 000
2680819	SUCLG2	NM_003848 // SUC	CCACTGTCC	2.15934	chr12:94942021-94944325 (+) /	0.84396	1.79497	AA up vs CA	0006099 // tricarboxylic acid cycle // inferred from electronic annotation /// 000
2680819	SUCLG2	NM_003848 // SUC	GGACAGTAG	3.16539	chr12:94942021-94944325 (+) /	0.84396	1.79497	AA up vs CA	0006099 // tricarboxylic acid cycle // inferred from electronic annotation /// 000
2680819	SUCLG2	NM_003848 // SUC	CCACTGTCC	2.15934	chr3:67425142-67705002 (+) /	0.84396	1.79497	AA up vs CA	0006099 // tricarboxylic acid cycle // inferred from electronic annotation /// 000
2680819	SUCLG2	NM_003848 // SUC	GGACAGTAG	3.16539	chr3:67425142-67705002 (+) /	0.84396	1.79497	AA up vs CA	0006099 // tricarboxylic acid cycle // inferred from electronic annotation /// 000
3608638	SV2B	NM_014848 // SV2	GGACACTGG	2.8087	chr15:91769329-91838514 (+) /	-1.09783	-2.1403	AA down vs CA	0006810 // transport // inferred from electronic annotation /// 0006836 // neur

3608638	SV2B	NM_014848 // SV2	CCAGTGTCC	2.8087	chr15:91769329-91838514 (+) /	-1.09783	-2.1403	AA down vs CA	0006810 // transport // inferred from electronic annotation /// 0006836 // neur
2560524	TACR1	NM_001058 // TAC	GCCCTGTCC	4.94793	chr2:75278162-75426183 (-) //	-1.07546	-2.1074	AA down vs CA	0002118 // aggressive behavior // inferred from electronic annotation /// 00025
2560524	TACR1	NM_001058 // TAC	AAAGTGTCC	3.38289	chr2:75278162-75426183 (-) //	-1.07546	-2.1074	AA down vs CA	0002118 // aggressive behavior // inferred from electronic annotation /// 00025
2560524	TACR1	NM_001058 // TAC	TGATTGTCC	2.26764	chr2:75278162-75426183 (-) //	-1.07546	-2.1074	AA down vs CA	0002118 // aggressive behavior // inferred from electronic annotation /// 00025
2560524	TACR1	NM_001058 // TAC	GCCCTGTCC	4.94793	chr2:75278162-75426183 (-) //	-1.07546	-2.1074	AA down vs CA	0002118 // aggressive behavior // inferred from electronic annotation /// 00025
2560524	TACR1	NM_001058 // TAC	AAAGTGTCC	3.38289	chr2:75278162-75426183 (-) //	-1.07546	-2.1074	AA down vs CA	0002118 // aggressive behavior // inferred from electronic annotation /// 00025
2560524	TACR1	NM_001058 // TAC	TGATTGTCC	2.26764	chr2:75278162-75426183 (-) //	-1.07546	-2.1074	AA down vs CA	0002118 // aggressive behavior // inferred from electronic annotation /// 00025
2560524	TACR1	NM_001058 // TAC	GCCCTGTCC	4.94793	chr2:75276359-75426104 (-) //	-1.07546	-2.1074	AA down vs CA	0002118 // aggressive behavior // inferred from electronic annotation /// 00025
2560524	TACR1	NM_001058 // TAC	AAAGTGTCC	3.38289	chr2:75276359-75426104 (-) //	-1.07546	-2.1074	AA down vs CA	0002118 // aggressive behavior // inferred from electronic annotation /// 00025
2560524	TACR1	NM_001058 // TAC	TGATTGTCC	2.26764	chr2:75276359-75426104 (-) //	-1.07546	-2.1074	AA down vs CA	0002118 // aggressive behavior // inferred from electronic annotation /// 00025
2950214	TAP1	NM_000593 // TAP	CCACTGTCC	2.15934	chr6:32812986-32821623 (-) //	0.922289	1.89512	AA up vs CA	0001916 // positive regulation of T cell mediated cytotoxicity // inferred from seq
2950214	TAP1	NM_000593 // TAP	GGACACTCT	3.13137	chr6:32812986-32821623 (-) //	0.922289	1.89512	AA up vs CA	0001916 // positive regulation of T cell mediated cytotoxicity // inferred from seq
2950214	TAP1	NM_000593 // TAP	GGACAGCGC	4.24108	chr6:32812986-32821623 (-) //	0.922289	1.89512	AA up vs CA	0001916 // positive regulation of T cell mediated cytotoxicity // inferred from seq
2805786	TARS	NM_152295 // TAR	GGACAATCA	2.26764	chr5:33441090-33468169 (+) /	0.871765	1.8299	AA up vs CA	0006412 // translation // non-traceable author statement /// 0006412 // transl
2805786	TARS	NM_152295 // TAR	GGATTGTCC	1.4039	chr5:33441090-33468169 (+) /	0.871765	1.8299	AA up vs CA	0006412 // translation // non-traceable author statement /// 0006412 // transl
2491386	TCF7L1	NM_031283 // TCF	CCAGTGTCC	2.8087	chr2:85360733-85537504 (+) /	-0.85984	-1.8148	AA down vs CA	0006325 // chromatin organization // non-traceable author statement /// 00063
3352847	TECTA	NM_005422 // TEC	GCCCTGTCC	4.94793	chr11:120973374-121061515 (-	-0.79403	-1.7339	AA down vs CA	0007160 // cell-matrix adhesion // inferred from electronic annotation /// 00076
3766716	TEX2	NM_018469 // TEX	GGACAGTGT	1.72747	chr17:62225049-62265688 (-) /	0.983335	1.97703	AA up vs CA	0006665 // sphingolipid metabolic process // non-traceable author statement ///
3766716	TEX2	NM_018469 // TEX	GGACAGTGT	1.72747	chr17:62225049-62265688 (-) /	0.983335	1.97703	AA up vs CA	0006665 // sphingolipid metabolic process // non-traceable author statement ///
3905145	TGM2	NM_004613 // TGM	TGACTGTCC	1.61828	chr20:36756862-36790002 (-) /	0.887736	1.85027	AA up vs CA	0001974 // blood vessel remodeling // inferred from electronic annotation /// 00
3905145	TGM2	NM_004613 // TGM	TGACTGTCC	1.61828	chr20:36766450-36793684 (-) /	0.887736	1.85027	AA up vs CA	0001974 // blood vessel remodeling // inferred from electronic annotation /// 00
3905145	TGM2	NM_004613 // TGM	TGACTGTCC	1.61828	chr20:36766351-36793646 (-) /	0.887736	1.85027	AA up vs CA	0001974 // blood vessel remodeling // inferred from electronic annotation /// 00
3905145	TGM2	NM_004613 // TGM	TGACTGTCC	1.61828	chr20:36765876-36768929 (-) /	0.887736	1.85027	AA up vs CA	0001974 // blood vessel remodeling // inferred from electronic annotation /// 00
2318416	THAP3	NM_138350 // THA	GGACAATTG	3.81475	chr1:6685143-6693622 (+) // 9	-0.98443	-1.9785	AA down vs CA	---
2318416	THAP3	NM_138350 // THA	GGACAATTG	3.81475	chr1:6684956-6685457 (+) // 1	-0.98443	-1.9785	AA down vs CA	---
2679796	THOC7	NM_025075 // THC	GGACAGTCA	1.61828	chr3:63819546-63825450 (-) //	0.804706	1.74679	AA up vs CA	0006397 // mRNA processing // inferred from electronic annotation /// 0006406
2333051	TIE1	NM_005424 // TIE1	ACAATGTCC	2.59328	chr1:43766707-43788778 (+) /	-0.89682	-1.862	AA down vs CA	0001570 // vasculogenesis // inferred from electronic annotation /// 0001701 /
2489385	TLX2	NM_016170 // TLX	GGACAGAGC	4.24108	chr2:74741933-74743361 (+) /	-0.9934	-1.9909	AA down vs CA	0001707 // mesoderm formation // inferred from electronic annotation /// 0006
2489385	TLX2	NM_016170 // TLX	GGACAGAGC	4.24108	chr2:74741610-74743890 (+) /	-0.9934	-1.9909	AA down vs CA	0001707 // mesoderm formation // inferred from electronic annotation /// 0006
3261723	TMEM18	NM_024789 // TME	CCAGTGTCC	2.8087	chr10:104230746-104236800 (-	-0.90174	-1.8683	AA down vs CA	---
2573232	TMEM18	NR_000034 // TME	GGTCTGTCC	4.99562	chr2:120979091-120980954 (-)	0.960111	1.94546	AA up vs CA	---
3089816	TNFRSF1	NM_003841 // TNF	GGACACTTT	3.38289	chr8:22960498-22974950 (+) /	-0.78874	-1.7276	AA down vs CA	0006915 // apoptosis // inferred from electronic annotation /// 0007165 // sign
3089816	TNFRSF1	NM_003841 // TNF	GGACACTTT	3.38289	chr8:22960442-22974683 (+) /	-0.78874	-1.7276	AA down vs CA	0006915 // apoptosis // inferred from electronic annotation /// 0007165 // sign
2705706	TNFSF10	NM_003810 // TNF	GGACAGTTG	3.16539	chr3:172224175-172241264 (-)	0.994623	1.99256	AA up vs CA	0006915 // apoptosis // inferred from electronic annotation /// 0006917 // indu
2705706	TNFSF10	NM_003810 // TNF	GGACATTAT	3.59934	chr3:172224175-172241264 (-)	0.994623	1.99256	AA up vs CA	0006915 // apoptosis // inferred from electronic annotation /// 0006917 // indu
2705706	TNFSF10	NM_003810 // TNF	GGACAGTTG	3.16539	chr3:172223464-172241261 (-)	0.994623	1.99256	AA up vs CA	0006915 // apoptosis // inferred from electronic annotation /// 0006917 // indu

2705706	TNFSF10	NM_003810 // TNF	GGACATTAT	3.59934	chr3:172223464-172241261 (-)	0.994623	1.99256	AA up vs CA	0006915 // apoptosis // inferred from electronic annotation /// 0006917 // indu
2705706	TNFSF10	NM_003810 // TNF	GGACAGTTG	3.16539	chr3:172223300-172223725 (-)	0.994623	1.99256	AA up vs CA	0006915 // apoptosis // inferred from electronic annotation /// 0006917 // indu
2705706	TNFSF10	NM_003810 // TNF	GGACATTAT	3.59934	chr3:172223300-172223725 (-)	0.994623	1.99256	AA up vs CA	0006915 // apoptosis // inferred from electronic annotation /// 0006917 // indu
2444283	TNFSF4	NM_003326 // TNF	CCAGTGTCC	2.8087	chr1:173152873-173176351 (-)	-0.80742	-1.7501	AA down vs CA	0001816 // cytokine production // inferred from electronic annotation /// 00069
2641901	TRH	NM_007117 // TRH	CGAATGTCC	3.77969	chr3:129693562-129696780 (+)	-0.82706	-1.7741	AA down vs CA	0001666 // response to hypoxia // inferred from electronic annotation /// 00071
2641901	TRH	NM_007117 // TRH	AAATTGTCC	3.38289	chr3:129693562-129696780 (+)	-0.82706	-1.7741	AA down vs CA	0001666 // response to hypoxia // inferred from electronic annotation /// 00071
2641901	TRH	NM_007117 // TRH	GGACAAAGC	4.89044	chr3:129693562-129696780 (+)	-0.82706	-1.7741	AA down vs CA	0001666 // response to hypoxia // inferred from electronic annotation /// 00071
3844238	TRIM28	NM_005762 // TRIM	GGACACAGC	4.89044	chr19:59055868-59062083 (+) /	0.961764	1.94769	AA up vs CA	0000122 // negative regulation of transcription from RNA polymerase II promoter
3620799	TTBK2	NM_173500 // TTB	GGACACTCC	1.4039	chr15:43036545-43067601 (-) /	-0.96596	-1.9534	AA down vs CA	0006468 // protein phosphorylation // inferred from electronic annotation /// 00
2829562	TXNDC15	NM_024715 // TXN	GCTCTGTCC	4.24108	chr5:134210005-134235580 (+)	0.897426	1.86274	AA up vs CA	0045454 // cell redox homeostasis // inferred from electronic annotation
2829562	TXNDC15	NM_024715 // TXN	GGACACTGT	2.37683	chr5:134210005-134235580 (+)	0.897426	1.86274	AA up vs CA	0045454 // cell redox homeostasis // inferred from electronic annotation
3373962	UBE2L6	NM_004223 // UBE	GGAGTGTCC	1.4039	chr11:57319136-57335131 (-) /	0.82326	1.7694	AA up vs CA	0006464 // protein modification process // traceable author statement /// 0019
3033924	UBE3C	NM_014671 // UBE	GGACAAAGC	4.89044	chr7:156931663-157062062 (+)	0.836724	1.78599	AA up vs CA	0000209 // protein polyubiquitination // inferred from direct assay /// 0006464
3857691	UQCRFS1	NM_006003 // UQC	GGACACTGA	1.5131	chr22:40271299-40272276 (+) /	0.897124	1.86235	AA up vs CA	0006810 // transport // inferred from electronic annotation /// 0009725 // resp
2731831	USO1	NM_003715 // USO	GGACAGTGG	2.15934	chr4:76649790-76735439 (+) //	0.826225	1.77304	AA up vs CA	0006810 // transport // inferred from electronic annotation /// 0006886 // intra
2731831	USO1	NM_003715 // USO	GGACAGTGG	2.15934	chr4:76649828-76735364 (+) //	0.826225	1.77304	AA up vs CA	0006810 // transport // inferred from electronic annotation /// 0006886 // intra
3064462	VGF	NM_003378 // VGF	GGACAGCGC	4.24108	chr7:100805796-100808852 (-)	-0.78382	-1.7217	AA down vs CA	0001541 // ovarian follicle development // inferred from electronic annotation ///
3375147	VPS37C	NM_017966 // VPS	GGACACTTC	1.65542	chr11:60897731-60899007 (-) /	-1.10563	-2.1519	AA down vs CA	0006810 // transport // inferred from electronic annotation /// 0015031 // prot
3579546	WARS	NM_004184 // WAI	GGACACTGG	2.8087	chr14:100800856-100841769 (-	1.139738	2.20341	AA up vs CA	0001525 // angiogenesis // inferred from electronic annotation /// 0006412 // t
3579546	WARS	NM_004184 // WAI	GGACACTGG	2.8087	chr14:100800127-100841769 (-	1.139738	2.20341	AA up vs CA	0001525 // angiogenesis // inferred from electronic annotation /// 0006412 // t
2354082	WDR3	NM_006784 // WDI	AGAATGTCC	3.34783	chr1:118475908-118503049 (+)	0.913362	1.88343	AA up vs CA	---
2807000	WDR70	NM_018034 // WDI	GGACACTGA	1.5131	chr5:37379469-37752772 (+) //	0.786922	1.72539	AA up vs CA	---
3376235	WDR74	NM_018093 // WDI	GGACAGTAC	1.00606	chr11:62600383-62607627 (-) /	0.839089	1.78892	AA up vs CA	---
3376235	WDR74	NM_018093 // WDI	GCACTGTCC	0	chr11:62600383-62607627 (-) /	0.839089	1.78892	AA up vs CA	---
3376235	WDR74	NM_018093 // WDI	GGACAGTAC	1.00606	chr11:62600383-62607053 (-) /	0.839089	1.78892	AA up vs CA	---
3376235	WDR74	NM_018093 // WDI	GCACTGTCC	0	chr11:62600383-62607053 (-) /	0.839089	1.78892	AA up vs CA	---
2427930	WDR77	NM_024102 // WDI	TAAGTGTCC	2.51915	chr1:111982604-111992067 (-)	0.79273	1.73235	AA up vs CA	0000387 // spliceosomal snRNP assembly // not recorded /// 0006357 // regula
2427930	WDR77	NM_024102 // WDI	TAAGTGTCC	2.51915	chr1:111982895-111991851 (-)	0.79273	1.73235	AA up vs CA	0000387 // spliceosomal snRNP assembly // not recorded /// 0006357 // regula
2405192	YARS	NM_003680 // YAR	GGACACCGC	4.89044	chr1:33240841-33282921 (-) //	0.823097	1.7692	AA up vs CA	0006412 // translation // inferred from electronic annotation /// 0006418 // tRN
2577644	YSK4	NM_025052 // YSK	AGACTGTCC	2.48201	chr2:135737231-135739222 (-)	-0.78244	-1.72	AA down vs CA	0006468 // protein phosphorylation // inferred from electronic annotation
2375810	ZC3H11A	NM_014827 // ZC3	GGACAATGT	2.37683	chr1:203786051-203823246 (+)	0.818131	1.76312	AA up vs CA	---
2375810	ZC3H11A	NM_014827 // ZC3	GGACAATGT	2.37683	chr1:203786051-203823246 (+)	0.818131	1.76312	AA up vs CA	---
3221277	ZFP37	NM_003408 // ZFP:	TAATTGTCC	2.51915	chr9:115804173-115818996 (-)	-0.93697	-1.9145	AA down vs CA	0006350 // transcription // inferred from electronic annotation /// 0006355 // r
3221277	ZFP37	NM_003408 // ZFP:	GGACATTCA	2.48409	chr9:115804173-115818996 (-)	-0.93697	-1.9145	AA down vs CA	0006350 // transcription // inferred from electronic annotation /// 0006355 // r
3976240	ZNF157	NM_003446 // ZNF	ATACTGTCC	2.73353	chrX:47229981-47273355 (+) //	-0.83685	-1.7861	AA down vs CA	0000122 // negative regulation of transcription from RNA polymerase II promoter
3856646	ZNF208	NM_007153 // ZNF	GGACAGAGC	4.24108	chr19:22153484-22171712 (-) /	-1.06695	-2.095	AA down vs CA	0006350 // transcription // inferred from electronic annotation /// 0006355 // r

3843180	ZNF304	NM_020657 // ZNF	GCATTGTCC	0.64936	chr19:57862674-57871041 (+) ,	-0.87298	-1.8314	AA down vs CA	0006350 // transcription // inferred from electronic annotation /// 0006355 // r
3843180	ZNF304	NM_020657 // ZNF	GGACATTCA	2.48409	chr19:57862674-57871041 (+) ,	-0.87298	-1.8314	AA down vs CA	0006350 // transcription // inferred from electronic annotation /// 0006355 // r
3844152	ZNF324	NM_014347 // ZNF	GGACATTGA	1.72955	chr19:58978462-58984765 (+) ,	-1.04363	-2.0614	AA down vs CA	0006350 // transcription // inferred from electronic annotation /// 0006355 // r
3159013	ZNF34	NM_030580 // ZNF	GGACACTGG	2.8087	chr8:145998507-146012691 (-)	-0.9103	-1.8794	AA down vs CA	0006350 // transcription // inferred from electronic annotation /// 0006355 // r
3851293	ZNF44	NM_001164276 //	GGACAGTGA	0.86373	chr19:12359217-12405649 (-) /	-0.83582	-1.7849	AA down vs CA	0006350 // transcription // inferred from electronic annotation /// 0006355 // r
3851293	ZNF44	NM_001164276 //	GGACAGTGA	0.86373	chr19:12382828-12384007 (-) /	-0.83582	-1.7849	AA down vs CA	0006350 // transcription // inferred from electronic annotation /// 0006355 // r
3688178	ZNF668	NM_001172669 //	GAAGTGTCC	1.65542	chr16:31072163-31075683 (-) /	-0.92472	-1.8983	AA down vs CA	0006350 // transcription // inferred from electronic annotation /// 0045449 // r
3688178	ZNF668	NM_001172669 //	GGACACTTC	1.65542	chr16:31072163-31075683 (-) /	-0.92472	-1.8983	AA down vs CA	0006350 // transcription // inferred from electronic annotation /// 0045449 // r
3119516	ZNF696	NM_030895 // ZNF	GGACATTTA	2.73561	chr8:144373595-144380164 (+)	-0.82203	-1.7679	AA down vs CA	0006350 // transcription // inferred from electronic annotation /// 0045449 // r
3983154	ZNF711	NM_021998 // ZNF	TTAGTGTCC	2.51915	chrX:84499089-84526927 (+) //	-0.8571	-1.8114	AA down vs CA	0006350 // transcription // inferred from electronic annotation /// 0045449 // r
3983154	ZNF711	NM_021998 // ZNF	TCACTGTCC	0.86373	chrX:84499089-84526927 (+) //	-0.8571	-1.8114	AA down vs CA	0006350 // transcription // inferred from electronic annotation /// 0045449 // r
2689452	ZNF80	NM_007136 // ZNF	GGACACTCT	3.13137	chr3:113953477-113956235 (-)	-0.86959	-1.8271	AA down vs CA	0006350 // transcription // inferred from electronic annotation /// 0006355 // r
2689452	ZNF80	NM_007136 // ZNF	GGACACTCT	3.13137	chr3:113953477-113956235 (-)	-0.86959	-1.8271	AA down vs CA	0006350 // transcription // inferred from electronic annotation /// 0006355 // r
